# ΔNp63α expression induces loss of cell adhesion in triple-negative breast cancer cells

**DOI:** 10.1186/s12885-016-2808-x

**Published:** 2016-10-10

**Authors:** Marta Nekulova, Jitka Holcakova, Xiaolian Gu, Vaclav Hrabal, Sotiris Galtsidis, Paulina Orzol, Yajing Liu, Stella Logotheti, Vassilis Zoumpourlis, Karin Nylander, Philip J Coates, Borivoj Vojtesek

**Affiliations:** 1 Regional Centre for Applied Molecular Oncology, Masaryk Memorial Cancer Institute, Zluty kopec 7, Brno, 65653 Czech Republic; 2 Department of Medical Biosciences, Umeå University, Umeå, 90185 Sweden; 3 Institute of Biology, Medicinal Chemistry & Biotechnology, NHRF, Athens, Greece; 4 NCRC, 026-329S, University of Michigan, Ann Arbor, MI 48109 USA

**Keywords:** p63 isoforms, Triple-negative breast cancer, Adhesion

## Abstract

**Background:**

p63, a member of the p53 protein family, plays key roles in epithelial development and carcinogenesis. In breast cancer, p63 expression has been found predominantly in basal-A (epithelial-type) triple-negative breast carcinomas (TNBC). To investigate the functional role of p63 in basal-A TNBC, we created MDA-MB-468 cell lines with inducible expression of the two major N-terminal p63 isoforms, TAp63α and ∆Np63α.

**Results:**

TAp63α did not have significant effect on gene expression profile and cell phenotype, whilst the main effect of ΔNp63α was reduction of cell adhesion. Gene expression profiling revealed genes involved in cell adhesion and migration whose expression relies on overexpression of ΔNp63α. Reduced cell adhesion also led to decreased cell proliferation in vitro and in vivo. Similar data were obtained in another basal-A cell line, BT-20, but not in BT-549 basal-B (mesenchymal-like) TNBC cells.

**Conclusions:**

In basal-A TNBC cells, ∆Np63α has much stronger effects on gene expression than TAp63α. Although p63 is mentioned mostly in connection with breast cell differentiation and stem cell regulation, we showed that a major effect of p63 is regulation of cell adhesion, a process important in metastasis and invasion of tumour cells. That this effect is not seen in mesenchymal-type TNBC cells suggests lineage-dependent functions, mirroring the expression of ∆Np63α in primary human breast cancers.

**Electronic supplementary material:**

The online version of this article (doi:10.1186/s12885-016-2808-x) contains supplementary material, which is available to authorized users.

## Background

p63 is a member of the p53 family of transcription factors and is known to be involved in the regulation of epithelial development and carcinogenesis. The *TP63* gene is expressed as a spectrum of protein isoforms due to alternative promoter usage and alternative splicing at the 3´ end of the transcript [1]. There are two N-terminal protein isoforms: TAp63, containing a p53-like N-terminal transactivation domain, and ΔNp63, the N-terminally truncated isoform that lacks this transactivation domain. ΔNp63 was originally thought to be only a dominant negative inhibitor that blocks the function of full-length p53/p63/p73 proteins. Later it was found that ΔNp63 also transactivates target genes due to the presence of alternative transactivation domains [2, 3] and that it is the predominant isoform in most normal adult tissues according to immunohistochemical studies [4, 5]. Compared to ΔNp63, TAp63 is expressed as the main isoform only in specific cell types such as germ cells and B-lymphocytes [4, 6, 7]. Similarly, ΔNp63 is overexpressed in many cancers, especially squamous carcinomas [5, 8] in contrast to TAp63 which is usually detected in tumour tissue at low level excepting B-cell lymphomas [5, 9].

In normal breast tissue, ΔNp63 expression is restricted to basal/myoepithelial cells [5, 10, 11] and p63 is essential for mammary gland morphogenesis during embryonic development [12]. In adulthood, ΔNp63 is important for maintenance of basal cell characteristics of breast epithelial cells [13], for correct luminal cell proliferation and differentiation during lactation when it regulates paracrine basal-to-luminal cell signalling [14], and as a pro-survival factor of multipotent progenitor cells during post-lactational involution [15]. ΔNp63 expression is also linked with mammary stem cells – in mammary tissue ΔNp63 is expressed in the basal cell layer which is thought to contain stem cells [16], its expression was detected in activated stem cells isolated from developing mouse mammary tissue [17] and in stem cells isolated from mouse mammary epithelial cell line [18]. Moreover, Thomas et al. have isolated p63-positive stem cell-like multi-potent cells from breast milk [19] and Li et al. identified reciprocal interactions between p63 isoforms and hedgehog signalling in mammary stem and progenitor cells that regulate initiation and progression of the mammary regenerative cycle. In this situation, ΔNp63 blocks and TAp63 promotes differentiation along the luminal lineage [20].

In breast cancer, ΔNp63 is highly expressed in a subset of tumours with metaplastic and basal-like features that are frequently triple-negative [21–24]. Triple-negative breast cancers (TNBC) are defined by lack of estrogen receptor (ER), progesterone receptor (PR) and human epidermal growth factor receptor 2 (HER2). TNBC are highly proliferative, biologically more aggressive and exhibit poor prognosis compared to other types of breast cancer [25, 26]. With no targeted treatments currently available, patients with TNBC have a high risk of relapse and shorter overall survival compared to other breast cancer subtypes [27].

Concerning the role of p63 in breast cancer cells, ΔNp63 has been proposed as a pro-tumourigenic transcription factor that promotes cancer stem cell (CSC) features [21]. Consistent with this notion, ΔNp63 promotes normal mammary stem cell activity by enhancement of Wnt signalling and through this mechanism governs tumour-initiating activity of basal-like breast cancer [28]. In general agreement with these findings, abrogation of endogenous ΔNp63 causes a switch towards luminal phenotype and away from the basal phenotype in basal breast cancer cells, indicating a role in lineage regulation, although p63 silencing was insufficient to cause full luminal-type differentiation [29]. Further, ΔNp63 acts as a survival factor in a subset of breast cancers by antagonizing p73-mediated apoptosis [23]. In contrast, Buckley et al. have shown that ΔNp63 cooperates with BRCA1 to regulate growth control and maintain genomic stability in normal breast cells [30]. They also suggested that defects in BRCA1-ΔNp63 signalling are key events in the pathogenesis of basal-like breast cancer. In agreement with this idea, ΔNp63 and ΔNp73 up-regulate key DNA damage repair proteins (BRCA2, RAD50, RAD51, mre11, ATM) [31, 32] and loss of p63/p73 promotes mammary tumour formation in mice [33]. p63 has also been shown to play a tumour suppressor role in breast cancers because abrogation of its function through interaction with mutant p53-SMAD complex led to enhanced metastasis [34].

In view of the wide-ranging roles and effects of p63 that have been proposed in normal breast and TNBC, definitive roles for ΔNp63 and TAp63 in basal-type breast cancer need to be clarified. In our work we further elucidated the role of these two p63 isoforms in TNBC cells.

## Methods

### Cell culture conditions, vectors and transfections, beta-galactosidase assay

MDA-MB-468, BT-20 and BT-549 breast carcinoma cells were obtained from ATCC and maintained in Dulbecco’s modified Eagle’s medium (DMEM) supplemented with 10 % fetal bovine serum (HyClone, Thermo Scientific, USA), 1 mM sodium pyruvate (Sigma Aldrich, USA) and penicillin/streptomycin (HyClone, Thermo Scientific, USA) at 37 °C in CO_2_ incubator in humidified atmosphere.

To generate clones with inducible expression of ΔNp63α and TAp63α, MDA-MB-468 cells were transfected with pcDNA6/TR (Life Technologies, USA) vector and selected with blasticidin (5 μg/ml) for three weeks. Resistant colonies were picked and expanded for further analysis under selective conditions. Beta-galactosidase assay was used to test the inducibility of stable transfectants. MDA-MB-468-pcDNA6/TR cells were transiently transfected with pT-REx/GW-30/lacZ vector (Life Technologies, USA), lacZ expression was induced by incubation in DMEM with 1 μg/ml tetracycline for 24 h. Cells were fixed in PBS supplemented with 5 mM EDTA, 20 mM MgCl_2_ and 0,2 % glutaraldehyde for 10 min, washed in PBS and incubated in staining buffer (0,1 M phosphate buffer supplemented with 2 mM MgCl_2_, 5 mM potassium ferrocyanide, 5 mM potassium ferricyanide and 1 mg/ml X-gal substrate) for 30 min at room temperature. Development of blue colour was checked under light microscope. MDA-MB-468-pcDNA6/TR cells were then transfected with pT-REx-DEST30-ΔNp63α and pT-REx-DEST30-TAp63α vectors (containing full length cDNAs coding for human TAp63α and ΔNp63α; prepared from pT-REx-DEST30 using Gateway cloning technology, both from Life Technologies, USA) and transfected cultures were selected with G418 (500 μg/ml) for three weeks. Resistant colonies were picked, expanded under selective conditions and tested for inducibility of p63 expression by Western blot. pcDNA3-ΔNp63α and pcDNA3-GFP vectors were used for transient transfections of BT-20 and BT-549, cells were analysed 24 h after transfection. All transfections were performed using Amaxa Nucleofector Technology (Lonza, Switzerland) according to the manufacturer’s instructions.

### SDS-PAGE and Western blotting

Cells were harvested directly into lysis buffer (150 mM NaCl, 1 % Nonidet P-40, 50 mM Tris-HCl pH 8.0, 5 mM EDTA pH 8.0, protease inhibitor cocktail) and 20 μg of total protein in NuPAGE LDS Sample Buffer (Life Technologies, USA) loaded on 10 % polyacrylamide gels, separated and transferred onto nitrocellulose membranes in Mini-PROTEAN Electrophoresis System (Bio-Rad, USA) for 90 min applying 100 V in transfer buffer (240 mM Tris, 190 mM glycine, 20 % methanol). Membranes were blocked in 5 % non-fat milk in PBS with 0.1 % Tween and incubated overnight with primary antibodies at 4 °C. Primary antibodies used: anti-p63 clone SFI-6 (DCS Innovative Diagnostik-Systeme, Germany), anti-TAp63 clone TAp63-4.1 and anti-ΔNp63 clone ΔNp63-1.1 and polyclonal ΔNp63-44 [4] and anti-actin AC40 (Sigma-Aldrich, USA). Membranes were then incubated with appropriate HRP-conjugated secondary antibodies (RAM-Px, SWAR-Px, Dako, Denmark) diluted 1 : 1000 for 1 h at room temperature. Signal was detected using ECL reagent (Amersham Pharmacia Biotech, UK).

### Gene expression profiling

MDA-MB-468 cells were cultured with 1 μg/ml tetracycline for 24 h and harvested. Total RNA was isolated using TRI Reagent (Sigma-Aldrich, USA) and 1-bromo-3-chloropropane, precipitated with ethanol and diluted in RNAse-free water. Total RNA was labelled using TargetAmp Nano Labeling Kit for Illumina Expression BeadChip (Illumina, USA) and subsequently used for genome-wide expression profiling using HumanHT-12 v4 Expression BeadChip Kit (Illumina, USA). Results were analysed using R (The R Project for Statistical Computing), LIMMA software package [35], MultiExperiment Viewer [36] and GenomeStudio (Illumina, USA). Web tools from DAVID Bioinformatics Resources 6.7 [37] were employed to analyse gene ontology. The experiment was performed in three biological replicates.

### Quantitative RT-PCR

For analysis of individual gene expression, cells were grown for 24 h in media with 1 μg/ml tetracycline. Total RNA was isolated using RNeasy Mini Kit (Qiagen, Netherlands). 1 μg of total RNA was converted into cDNA using RevertAid H Minus Reverse Transcriptase (Thermo Fisher Scientific, USA). Quantitative RT-PCR was performed in technical triplicates using ABI 7900HT Fast Real Time PCR System (Applied Biosystems, USA) and repeated twice. Relative quantification of gene expression was performed in SDS 2.3 and RQ Manager 1.2 software (Applied Biosystems, USA) and RealTime StatMiner (Integromics, Spain). Primers are listed in Additional file 1.

### xCELLigence assay

xCELLigence system (ACEA Biosciences, USA) was used for real-time analysis of cell adhesion and proliferation. Before cells were seeded, background was measured 45 min after adding 50 μl media into each well of E-plate 16. Cells were trypsinized, resuspended in DMEM, counted using haemocytometer and 20 000 cells in 100 μl added per well. Impedance signal, which depends on the coverage of the electrodes with cells, was measured for 48 h in 15 min intervals. Tetracycline (1 μg/ml) was added at the beginning of the experiment (time 0). Two independent experiments were performed, each sample was in triplicate.

### Cell detachment and wound-healing assays

For cell detachment assay, cells were grown in 96-well plate for 24 h and then treated with 1 μg/ml tetracycline to induce p63 expression for 24 or 48 h. After washing in 0.5 % EGTA in PBS, cells were incubated in trypsin solution (0.025 % trypsin and 0.5 % EGTA in PBS) for the indicated periods of time. Trypsinization was stopped by adding 100 μl DMEM with 10 % FBS. Cells were then washed in PBS, fixed in 4 % paraformaldehyde for 10 min, washed again in PBS and stained with crystal violet (5 mg/ml). After washing in tap water, cells were dried for 30 min at RT and then 2 % SDS was used to dissolve the colour. Absorbance was measured at 595 nm. Three independent experiments were performed, each sample was in hexaplicate.

For wound healing assay, cells were seeded in 6-well plates and cultured until confluent. Scratches were made using pipette tips and phase contrast images were taken with Eclipse Ti-E inverted microscope system (Nikon, Japan) for 48 h at 1 h intervals.

### Cell proliferation assay, viability assay and cell cycle analysis

To compare cell proliferation, cells were grown for 24 h in 6-well plates and then treated with 1 μg/ml tetracycline to induce p63 expression. Both adherent and detached cells were harvested into 2 ml of DMEM 0, 24, 48 and 72 h after tetracycline treatment and counted in haemocytometer. Three independent experiments were performed.

Propidium Iodide (PI) exclusion assay was used for measurement of cell viability after 0, 24, 48 and 72 h treatment with 1 μg/ml tetracycline. Medium containing floating cells was removed and attached cells were trypsinized (efficiency of trypsinization was checked under the light microscope) and pooled with the floating cells. After addition of PI (1 μg/ml final concentration) cells were analysed for red fluorescence by flow cytometry (FACSVerse, BD Biosciences, USA). The percentage of PI positive (dead) cells was determined, after exclusion of cell debris, using FACSuite software (BD Biosciences, USA). Two independent biological experiments were performed, each sample was in triplicate.

For cell cycle analysis, cells were treated with 1 μg/ml tetracycline for 24 h, trypsinized, washed in PBS and fixed in 70 % ethanol for 2 h on ice. After washing in PBS, cells were resuspended in 1 ml of PI staining solution (0.1 % Triton X-100, 10 μg/ml PI, 100 μg/ml RNase A in PBS) and incubated at RT in the dark for 30 min. Cell cycle analysis was performed with FACSVerse using FACSuite software (both BD Biosciences, USA) and ModFit LT 4.0 software (Verity Software House, USA). Two independent experiments were performed, each sample was in triplicate.

### Tumour xenografts

10^6^ cells were resuspended in 100 μl PBS and injected subcutaneously into the left and right flanks of 6-week old female SCID mice. Mice were divided in 3 groups (MDA-MB-468 parental, MDA-MB-468-ΔNp63α untreated and MDA-MB-468-ΔNp63α tetracycline treated). Tumours were allowed to grow for 3 weeks after the injections until tumour onset in all injected sites. Then, the group of MDA-MB-468-ΔNp63α tetracycline treated mice were orally gavaged with 0.2 ml of tetracycline solution 10 mg/ml to induce ΔNp63 expression, on a daily basis for the next 8 weeks. During this period tumour volumes were measured using callipers and calculated using the formula ½ x height x width x length. At the end of the observation period, mice were sacrificed, tumours excised and photographed.

## Results

### Stably transfected cell lines with inducible expression of p63 isoforms

**Fig. 1:**
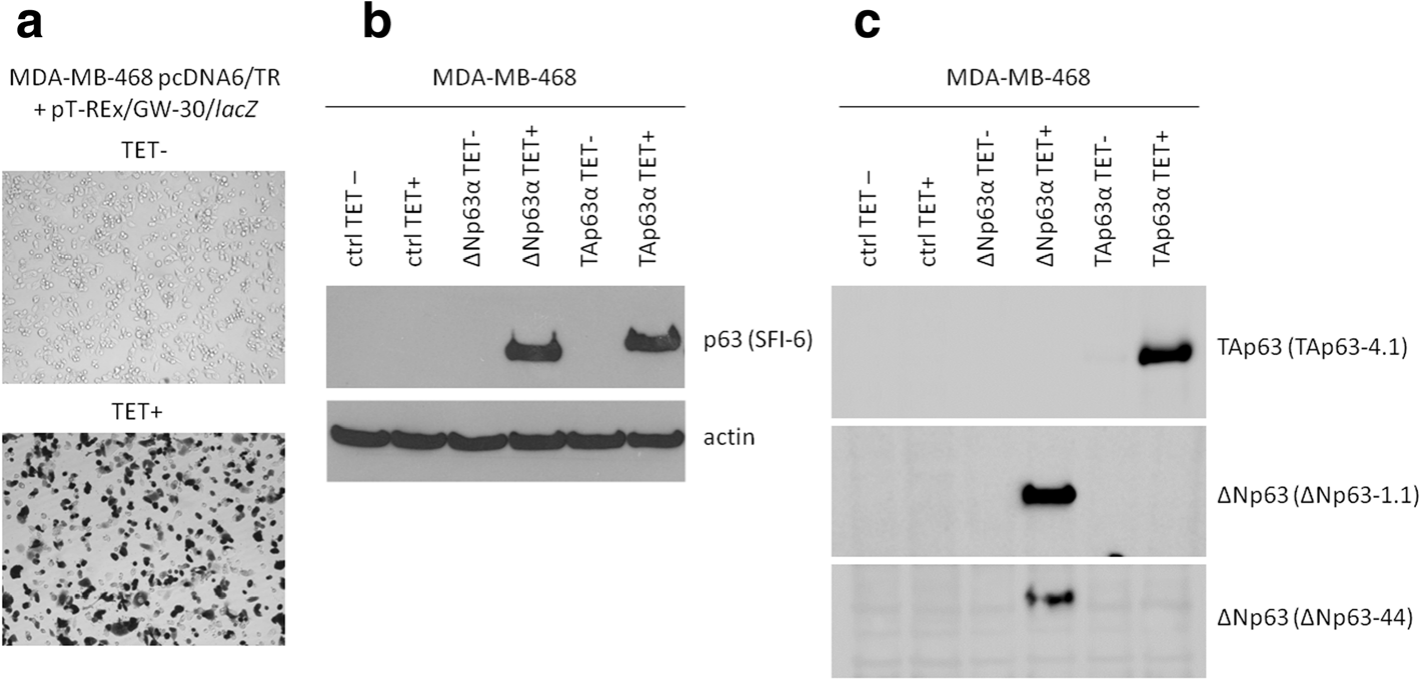
MDA-MB-468 cells with inducible expression of p63 isoforms. **a** MDA-MB-468-pcDNA6/TR clone with stable expression of tetracycline repressor transiently transfected with vector encoding beta-galactosidase under control of tetracycline-inducible promoter; staining of beta-galactosidase activity 24 h after tetracycline (TET) treatment; **b**, **c** Western blots of TAp63α and ΔNp63α in MDA-MB-468-ΔNp63α and MDA-MB-468-TAp63α cells 24 h after tetracycline treatment; MDA-MB-468-pcDNA6/TR parental clone was used as a negative control

To study the function of p63 in TNBC cells, we created an in vitro model that allows tight regulation of p63 expression. Because p63 is expressed predominantly in triple-negative and basal breast tumours with an epithelial phenotype (basal-A), MDA-MB-468 cell line was chosen as parental cell line. It has an ER-, PR-, HER- phenotype and belongs to basal-A subtype of breast tumours [38] and basal-like BL1 subgroup of TNBC [39]. MDA-MB-468 cells are reported to express negligible levels of p63 according to gene expression profiling, RNA sequencing, qRT-PCR and western blot [40–42], although one study identified an undefined level of TAp63 mRNA in these cells without identifying p63 protein [43]. Because cell lines may show different properties in different laboratories due to differences in culture conditions, we therefore characterized our MDA-MB-468 cells for expression of p63 isoforms by qRT-PCR and immunochemical approaches using antibodies specific for all isoforms and mono-specific antibodies for TAp63 and ΔNp63, showing a lack of all mRNA and p63 protein isoforms (Additional file 2). Clones stably transfected with tetracycline repressor were selected and their inducibility was subsequently tested by beta-galactosidase assay (Fig. 1a). Inducible ΔNp63α and TAp63α clones were subsequently prepared from this tetracycline-responsive clone by transfection with either pT-REx-DEST30-ΔNp63α or pT-REx-DEST30-TAp63α as described in the materials and methods section. Newly selected clones were tested for p63 isoform expression after tetracycline induction using the pan-p63 antibody SFI-6 that distinguishes the TA and ΔN isoforms due to their different molecular weights and the isoform specific p63 antibodies (Fig. 1b, c), and the two clones named MDA-MB-468-ΔNp63α and MDA-MB-468-TAp63α were used for subsequent experiments. The parental clone expressing only tetracycline-regulated inhibitor of transcription was named MDA-MB-468-pcDNA6/TR and used as negative control (Fig. 1 also shows that these cells do not contain detectable levels of endogenous p63).

### ΔNp63α regulates expression of adhesion-related genes in MDA-MB-468 cells

**Fig. 2:**
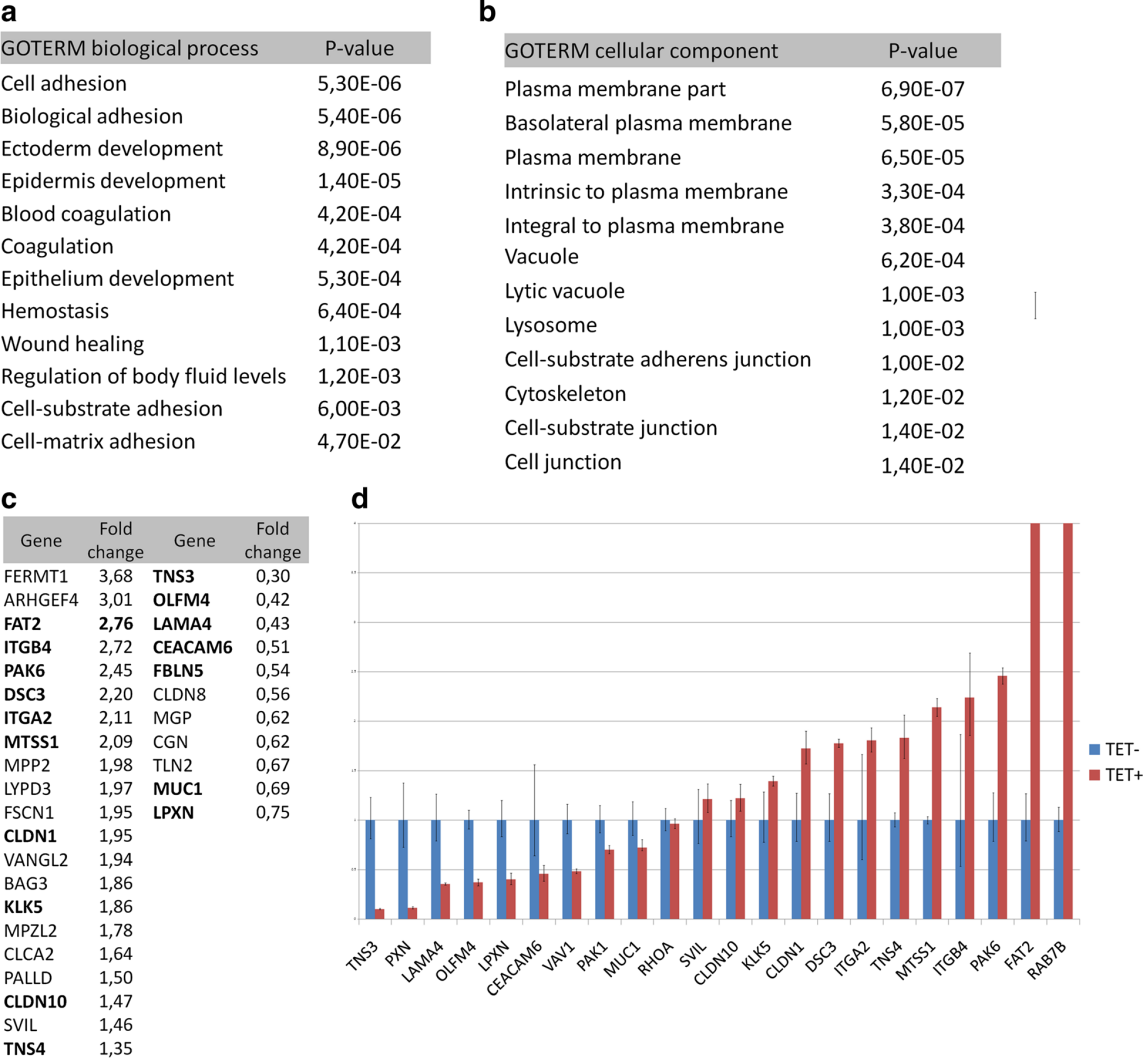
Selected results of gene expression profiling of MDA-MB-468-ΔNp63α cells 24 h after tetracycline (TET) treatment. **a** Analysis of gene ontology – most highly enriched biological processes. **b** Analysis of gene ontology – most highly enriched subcellular localizations of gene products. **c** Genes which were previously found to be involved in regulation of cell adhesion and migration and whose expression was significantly changed in MDA-MB-468-ΔNp63α cells. Expression of genes marked in bold was evaluated by qRT-PCR. **d** qRT-PCR analysis of selected p63-regulated genes in MDA-MB-468-ΔNp63α 24 h after tetracycline treatment

To map gene expression changes after induction of ∆Np63α and TAp63α, MDA-MB-468 cells were treated with tetracycline for 24 h and total RNA was used for genome-wide expression analysis. By this method we identified 428 genes whose expression changes significantly (adjusted p values < 0.05) after overexpression of ∆Np63α or TAp63α (Additional file 3). In both cases, *TP63* itself was the most highly up-regulated transcript and the identification of many previously reported p63 target genes (*S100A2*, *FGFR3*, *GPX2*, *MIR205*, *KRT5* etc.) in this dataset indicate good informational value and reliability of this analysis and also of the inducible system. Contrary to expectations that both ∆Np63α and TAp63α will affect gene expression, we found that only ∆Np63α significantly changed the gene expression profile, with TAp63 induction leading to up-regulation of only two genes, of which *TP63* itself is one. According to gene ontology (GO) analysis of the ∆Np63α-regulated genes, the most significantly enriched GO terms relating to biological processes were “cell adhesion” and “biological adhesion” (*p* < 0.001) (Fig. 2a). Other enriched adhesion-related GO terms were “cell-substrate adhesion” and “cell-matrix adhesion” (*p* < 0.05) indicating that cell-matrix adhesion was affected more than cell-cell adhesion. Among GO terms describing subcellular localization of the gene product, “plasma membrane” was the most enriched term (*p* < 0.001) (Fig. 2b). This is in agreement with the assumption that many adhesion-related proteins are transmembrane or membrane-associated. In accordance with this, many of the identified ∆Np63α-regulated genes have been previously found to be involved in cell adhesion, motility, cytoskeleton dynamics, migration and invasion (listed in Fig. 2c). This group included genes related to cell-extracellular matrix (ECM) adhesion and focal adhesions such as *ITGB4* (integrin beta 4), *ITGA2* (integrin alpha 2), *FERMT1* (fermitin/kindlin), *BAG3* (BCL2-associated athanogene 3), *KLK5* (kallikerin-5), *CLCA2* (chloride channel accessory 2), *PALLD* (palladin), *SVIL* (supervillin), *TNS4* (tensin-4), *TNS3* (tensin-3), *LPXN* (leupaxin), *TLN2* (talin-2) and *CEACAM6* (carcinoembryonic antigen-related cell adhesion molecule 6). Genes encoding proteins regulating cell-cell adhesion include *FAT2* (FAT tumour suppressor homolog 2), *DSC3* (desmocollin-3), *CLDN1* (claudin-1), *CLDN8* (claudin-8), *CLDN10* (claudin-10) and *CGN* (cingulin). Selected genes from this group were confirmed as ∆Np63α-regulated by qRT-PCR analysis (Fig. 2d). Interestingly, we observed an opposite regulation of tensins *TNS3* and *TNS4*, which are important for connection between cytoskeleton and integrins. It has been previously shown that inhibition of *TNS4* expression decreases cell migration, while inhibition of *TNS3* increases cell migration [44]. Moreover, *TNS4* expression correlated with metastasis in invasive breast carcinomas. According to our results, ∆Np63α increases *TNS4* and decreases *TNS3* expression. Among identified ∆Np63α-regulated genes there was also one gene encoding miRNA, miR-205, which is a known target of p63 in bladder [45] and prostate carcinomas [46] and which was shown to be down-regulated in TNBC [47] and suggested to play a tumour suppressor role in TNBC cells [48].

### ΔNp63α induces loss of adhesion and detachment of MDA-MB-468 cells

**Fig. 3:**
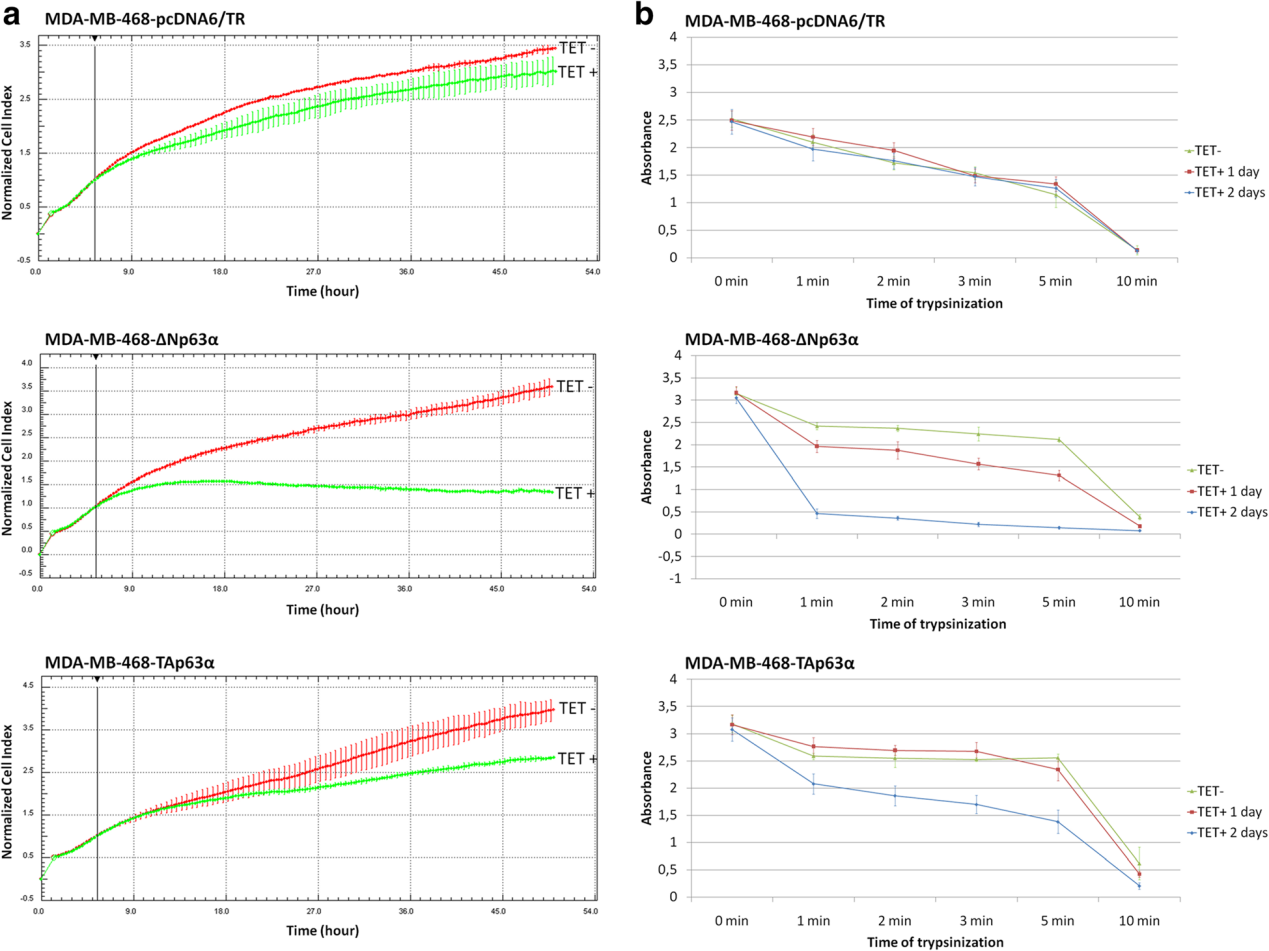
Analysis of MDA-MB-468-∆Np63α, MDA-MB-468-TAp63α and MDA-MB-468-pcDNA6/TR cell adhesion with xCELLigence system and detachment assay. **a** xCELLigence real-time cell analysis; tetracycline was added at the beginning of the experiment (time 0), decrease in cell index correlated with cell detachment; **b** detachment assay; decrease in absorbance correlated with cell sensitivity to trypsin and reduced cell adhesion

Since ΔNp63α changed expression of adhesion-related genes, we analysed adhesion of MDA-MB-468-ΔNp63α, MDA-MB-468-TAp63α and MDA-MB-468-pcDNA6/TR cells with and without tetracycline treatment. xCELLigence real-time cell analysis showed that cell-index values, which reflect the coverage of electrodes by cells, were significantly decreased after tetracycline treatment of MDA-MB-468-ΔNp63α and MDA-MB-468-TAp63α cells (Fig. 3a). Cell-index started to decrease approximately 9 h after addition of tetracycline and the impact of ∆Np63α was much higher compared to TAp63α. Tetracycline itself did not influence the cell-index (Fig. 3a).

This result was confirmed by detachment assay which measures cell adhesion based on cell sensitivity to trypsin [49] (Fig. 3b). Absorbance measured was dependent on the number of cells that remained adherent after trypsinization and thus correlated with cell adhesion. We observed strong decrease of absorbance after induction of ∆Np63α and a smaller decrease after TAp63α induction (Fig. 3b).

**Fig. 4:**
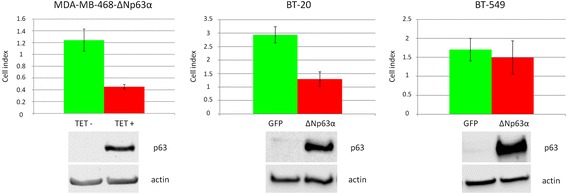
Different effects of ΔNp63α on cell adhesion/proliferation in BT-20 and BT-549 cells. BT-20 and BT-549 cells were transiently transfected with pcDNA3-ΔNp63α and pcDNA3-GFP plasmids and analyzed by xCELLigence 24 h after transfection. Cell index was significantly decreased in BT-20 cells expressing ΔNp63α (*p* < 0.01, *t*-test) and MDA-MB-468-ΔNp63α cells treated with tetracycline (*p* < 0.01, *t*-test) but not in BT-549 cells expressing ΔNp63α (*p* = 0.15, *t*-test) after 48 h. Expression of ΔNp63α 24 h after transfection / tetracycline treatment was confirmed by western blot

To confirm these data, we also used transient expression in another basal-A cell line, BT-20 and in a basal-B (mesenchymal) TNBC cell line, BT-549. Similar effects of ΔNp63α on cell adhesion/proliferation were observed when BT-20 cells were transiently transfected with ΔNp63α and cell index was measured by xCELLigence (Fig. 4). On the contrary, transient transfection of BT-549 did not significantly influence cell behaviour. These results suggest that ∆Np63α effect is not restricted to one cell line but depends on different molecular backgrounds.

### ΔNp63α-mediated loss of cell adhesion is accompanied by cell cycle arrest and reduced proliferation rate in vitro and in vivo

**Fig. 5:**
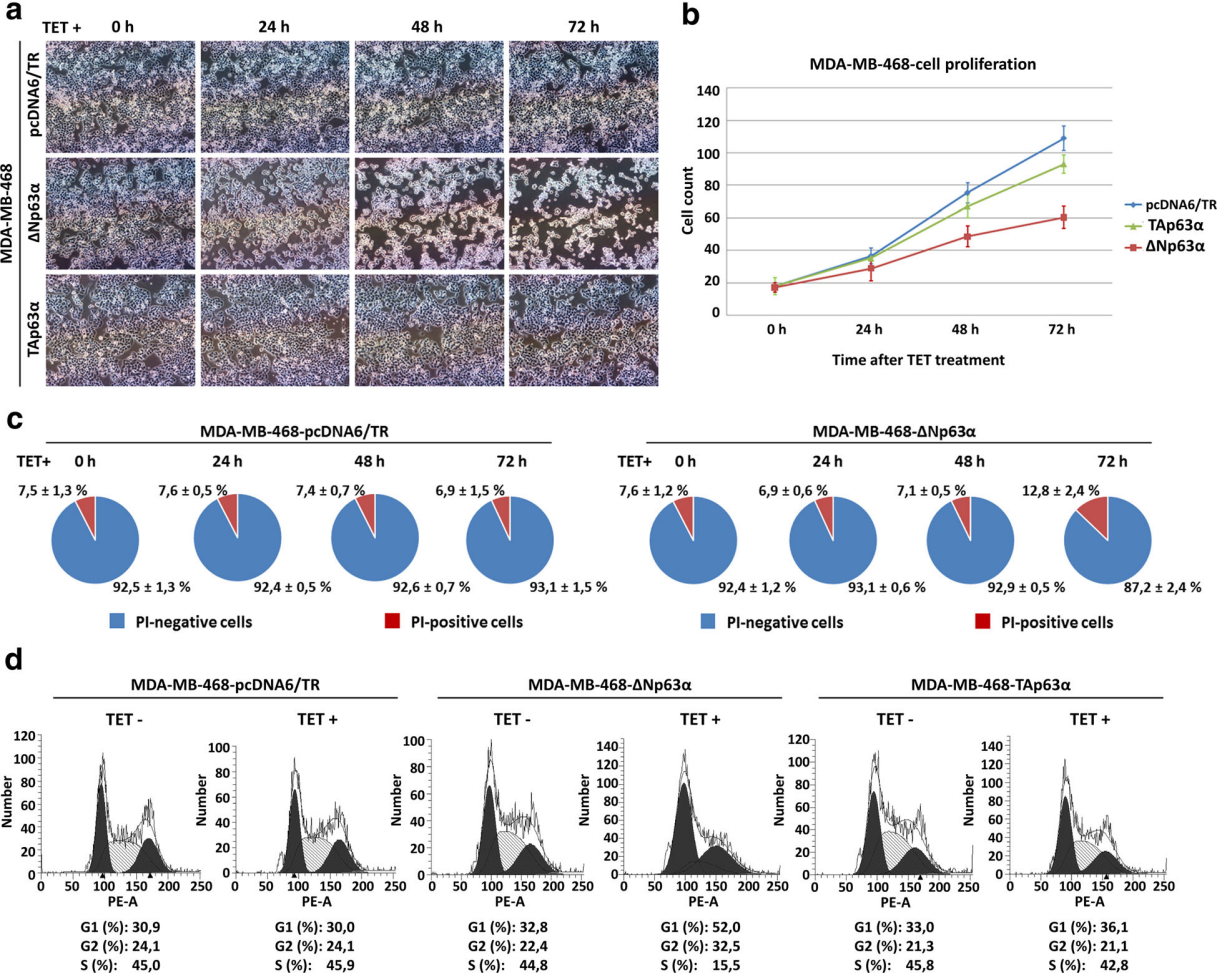
The effect of TAp63α and ΔNp63α expression on MDA-MB-468 cell growth, viability and cell cycle regulation.** a** Morphology of MDA-MB-468-ΔNp63α, MDA-MB-468-TAp63α and MDA-MB-468-pcDNA6/TR cells after tetracycline treatment; magnification 100×; **b** Cell proliferation of MDA-MB-468-ΔNp63α, MDA-MB-468-TAp63α and MDA-MB-468-pcDNA6/TR cells after tetracycline treatment (time 0); cells were counted in suspension by haemocytometer; **c** Flow cytometry-based cell viability assay using propidium iodide (PI), increased ratio of PI-positive cells correlates with decreased cell viability; **d** flow cytometry-based cell cycle analysis 24 h after tetracycline treatment

To investigate the effect of p63 isoforms on proliferation and viability of MDA-MB-468-ΔNp63α and MDA-MB-468-TAp63α cells, we detected their proliferation rate, propidium iodide (PI) exclusion and cell cycle regulation after tetracycline treatment. Adhesion and proliferation of MDA-MB-468-ΔNp63α were significantly decreased 48 and 72 h after tetracycline treatment compared to control cells, the effect of TAp63α was only weak (Fig. 5a, b).

**Fig. 6:**
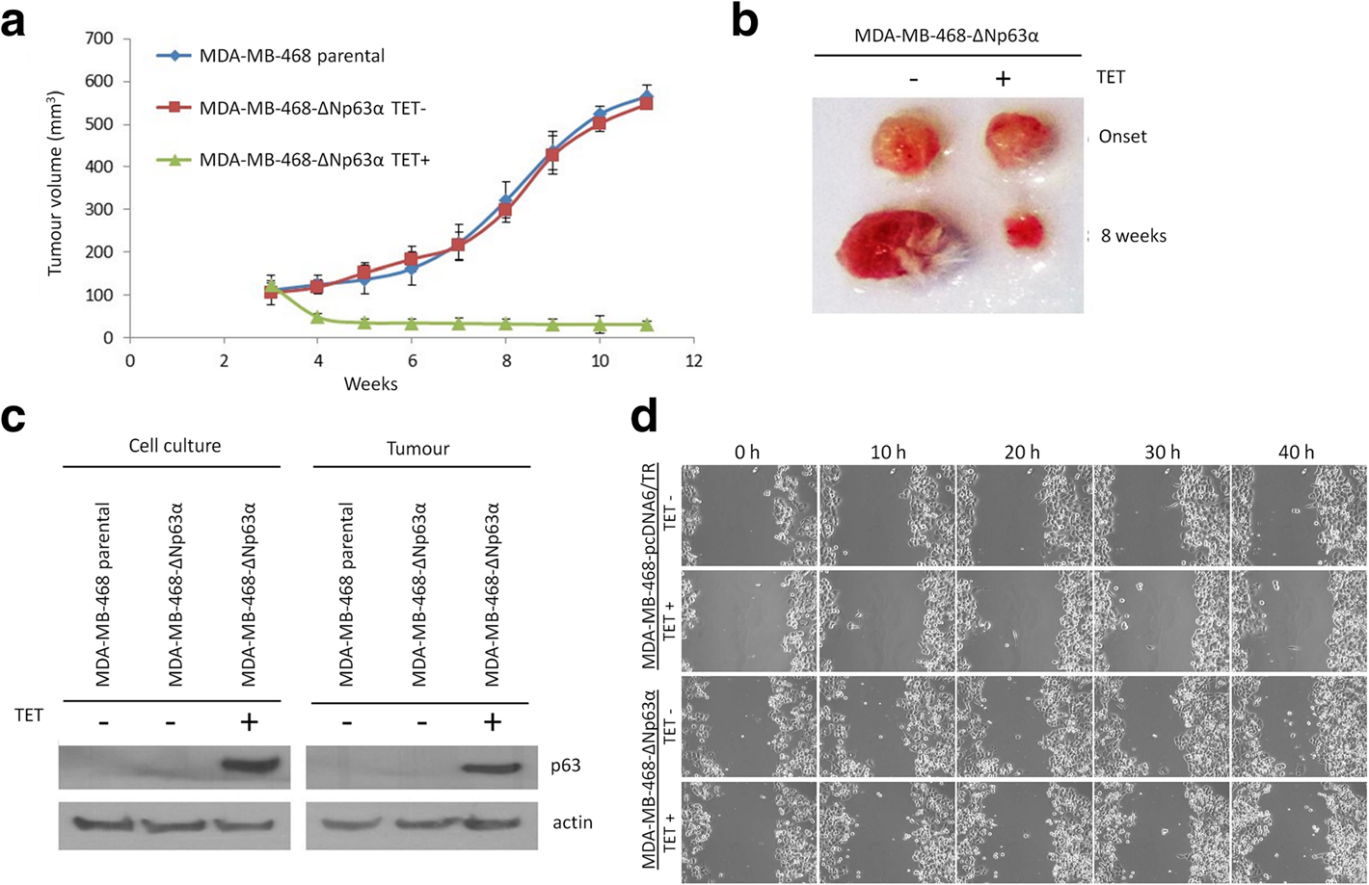
Induction of ΔNp63α in MDA-MB-468 cells reduces tumour size in vivo and does not change cell migration. **a** Growth rates of MDA-MB-468-ΔNp63α tumour xenografts in SCID mice (bars indicate standard deviation of tumour volumes, *n* = 6 sites injected); **b** representative images of tumours excised on tumour onset and after 8 weeks of ΔNp63α induction (experimental endpoint); **c** detection of p63 protein level in tumour lysates, protein extracts from corresponding cell cultures were used as controls; **d** representative images of wound-healing assay, tetracycline was added at the beginning of the experiment (time 0)

According to PI exclusion assay, cell viability of MDA-MB-468-ΔNp63α cells was not affected 24 and 48 h after tetracycline treatment, but the cells started to die 72 h after tetracycline treatment (Fig. 5c, increased number of PI positive cells, *p* < 0.05, *t*-test). Decreased proliferation of MDA-MB-468-ΔNp63α cells was accompanied by cell cycle arrest (Fig. 5d). To see whether these anti-proliferative effects of ΔNp63α expression are detectable also in vivo in more natural tumour environment, MDA-MB-468-ΔNp63α cells were subcutaneously injected into SCID mice. MDA-MB-468-ΔNp63α injected mice showed a significant decrease in tumour size after tetracycline treatment (*p* < 0.001, *t*-test) compared to the corresponding controls (Fig. 6a, b). Induction of ΔNp63α expression in MDA-MB-468 xenografts was confirmed by SDS-PAGE and western blot (Fig. 6c). Moreover, *GADD45A* and *CDKN1A* growth arrest-related genes were upregulated after ΔNp63α induction (Additional file 3). Because MDA-MB-468 cells are non-metastatic in xenografts [50], we cannot comment on whether p63 influenced this process, although there was no evidence of local invasion or overt distant metastasis in any group of mice.

Transwell migration assay with xCELLigence CIM plates and wound-healing assay were employed to study the effect of ∆Np63α on cell migration and invasion. MDA-MB-468 cells have very low migratory and invasive activity in vitro [38, 51] and transwell migration of these cells was not measurable. Wound-healing assay did not show any changes in cell migration after 20 h and evaluation of results after longer time was not possible due to changes in proliferation and cell detachment (Fig. 6d).

To compare resistance of MDA-MB-468 cells to anoikis, cells were grown in suspension on bacterial plates that did not allow them to adhere. Their viability was measured using PI exclusion assay after 24, 48 and 72 h. We did not found any apparent correlation between ∆Np63α expression and resistance to anoikis (Additional file 4).

## Discussion

TNBC is an aggressive form of breast cancer with shorter median time to relapse and death compared to other breast cancer subtypes, so there is an urgent need to expand our knowledge of TNBC biology and molecular mechanisms involved in tumour progression and metastasis [52]. p63 expression was previously detected in a group of breast tumours with triple-negative phenotype and basal-like epithelial features and the ∆Np63/TAp73 ratio shown to correlate with sensitivity to cisplatin treatment in vitro [23] as well as in TNBC patients [53]. To gain a deeper insight into the function of p63 in TNBC cells, we have created stably transfected MDA-MB-468 cell lines with inducible expression of ∆Np63α and TAp63α. MDA-MB-468 cells have a triple-negative phenotype and belong to the basal-A group of TNBC [39]. They show also several typical features of TNBC including mutated *TP53*, mutated *PTEN* and EGFR overexpression [54].

Although we could see similar levels of ∆Np63α and TAp63α induction at both the mRNA and protein levels, genome-wide expression profiling showed that only ∆Np63α has a significant impact on changes in gene expression. This is contrary to the initial assumptions that TAp63 is more active in transactivation of target genes compared to ∆N isoforms [1] and acts to induce apoptosis, growth arrest, and luminal differentiation in mammary epithelium [20, 55]. However, we and others have shown that ∆Np63 is the predominant isoform in adult epithelial tissues, including basal breast cells and basal-type breast cancers [4, 5, 28]. This latter paper also showed a negligible effect of TAp63 expression in breast epithelial cells, compared to a pro-tumorigenic role for∆Np63. The list of genes whose expression changed after ΔNp63α induction included many known target genes of p63 (see Results and Additional file 3), thus confirming the validity of our results [56–59].

Gene ontology analysis showed that ∆Np63α controls genes involved in regulating cell adhesion. Until recently, p63 was usually mentioned in connection with maintenance of basal and progenitor/stem cell phenotype in breast cancer cells or with cell survival and DNA damage responses [13, 20, 21, 28]. However, it has been previously demonstrated that p63 isoforms can regulate cell adhesion and related processes such as cell migration and invasion in different cancer types. p63 was shown to regulate adhesion and migration-related genes in head and neck squamous cell carcinoma cells and also in other squamous cell and urothelial carcinomas [60–62]. Moreover, knock-down of p63 caused down-regulation of cell adhesion-associated genes, cell detachment and anoikis in non-transformed mammary epithelial cells and keratinocytes [63]. CD44, a cell surface glycoprotein that regulates cell adhesion and migration and is connected to stem cell phenotype in different tumour types including breast cancer, was shown to be a direct ∆Np63 transcriptional target [64]. Most importantly, Cheung et al. have shown that invasion of breast tumours is led by a subpopulation of cells that are defined by their expression of basal epithelial genes including cytokeratin 14 (K14) and p63 [65]. Interestingly, although usually only a minority of cells within breast tumours expressed basal epithelial genes, knockdown of either K14 or p63 was sufficient to block collective invasion in primary tumour organoids. Although p63 is a transcriptional activator of K14, the authors suggested that p63 has K14-independent function in collective invasion. As changes in cell adhesion are an important event in the process of invasion and metastasis, we hypothesise that changes in expression of genes involved in regulation of cell adhesion that we have shown here could be a mechanism by which p63 influences cell invasion.

We recently reported results of CRISPR/Cas9-mediated *TP63* knock-out in the HCC1806 basal-A breast cancer cell line [29]. The major findings from this model were related to alterations of differentiation markers, where p63 is required to suppress expression of luminal markers and maintain the basal epithelial phenotype, but p63 loss is insufficient to induce full luminal-type differentiation. We also found that p63 depletion changed expression of adhesion molecules in a similar manner to that observed here, with reciprocal changes in *FAT2*, *ITGA2*, *CLDN1*, *LAMA4* and *CEACAM6* following p63 depletion [29] or p63 induction (this manuscript). However, either over-expression or gene knockout each caused a loss of cellular adhesion in the respective cell lines, indicating that the consequences of manipulating p63 levels are dependent on the cells under investigation, which likely relates to their dependence on p63 and their different genetic backgrounds.

The results of gene expression profiling were used to investigate cell phenotype, where induction of ∆Np63α caused visible loss of MDA-MB-468 cell adhesion and eventual cell detachment. Loss of cell adhesion was accompanied by decreased proliferation and cell cycle arrest and cell detachment caused decreased cell viability after 3 days of ∆Np63α induction. The same effect was observed in vivo in SCID mice, where induction of ∆Np63α correlated with decreased tumour size. This is contrary to the often reported importance of ∆Np63 for maintenance of cell proliferation [66–68] suggesting that p63 has a complex role in regulation of tumour cell fate. However, it is in agreement with previously reported ability of ∆Np63α to induce cell cycle arrest and upregulate *GADD45* and *CDKN1A* genes [69]. Similar data for the effects of ∆Np63α on proliferation and adhesion were seen in another basal-A (epithelial) type cell line, BT-20. Interestingly, these effects were not seen in basal-B (mesenchymal) type TNBC BT-549 cells, suggesting that ∆Np63α has cell context specific effects that relate to epithelial/mesenchymal cell phenotypes. This notion will require further investigation, but mirrors the expression pattern of p63 in primary human breast cancers, where p63 is a defining characteristic of basal-A TNBCs and basal-B mesenchymal-type TNBCs do not express p63 [38, 39].

## Conclusions

In summary, we have produced MDA-MB-468 basal-type breast cancer cells with inducible p63 expression vectors. Although most of our data are derived only from this cell line, we show that ΔNp63α had much stronger effects on gene expression than TAp63α, questioning whether this isoform has a physiological role in basal-type breast cancer. Further studies using other cell lines will be required to assess the generality of the findings and may indeed reveal further cell-context-dependent effects of p63 in cancer. In addition, correlations between p63 and the adhesion-related processes will be required in clinical samples to understand the potential role of p63 on patient outcome in basal-type breast cancer. In this respect, although p63 is usually mentioned mostly in connection with basal phenotype and stem cells in normal breast tissue and breast cancer, we showed that a major effect of p63 in MDA-MB-468 cells is regulation of cell adhesion, a process important in metastasis and invasion of tumour cells. We also found that ΔNp63α can inhibit cell proliferation in vitro and suppress tumour growth in vivo suggesting context-dependent effects of p63 in cancer.

## Additional files

Additional file 1: List of primers used for qRT-PCR. (PDF 266 kb)
Additional file 2: Expression of *TP63* in breast cancer cell lines. (PDF 407 kb)
Additional file 3: List of genes whose expression was changed 24 h after induction of ∆Np63α or TAp63α expression in MDA-MB-468 cells. (PDF 503 kb)
Additional file 4: Measurement of anoikis resistance of MDA-MB-468-∆Np63α cells. (PDF 249 kb)

## Abbreviations

CSC: cancer stem cells
ER: estrogen receptor
HER2: human epidermal growth factor receptor 2
PR: progesterone receptor
TET: tetracycline
TNBC: triple-negative breast carcinoma
