# Supplementary material for: ΔNp63α expression induces loss of cell adhesion in triple-negative breast cancer cells
Source: BMC Cancer. 2016 Oct 10;16:782. doi: 10.1186/s12885-016-2808-x (PMC5057421; doi:10.1186/s12885-016-2808-x)
Supplement: Additional file 1: — List of primers used for qRT-PCR. (PDF 266 kb) [file 12885_2016_2808_MOESM1_ESM.pdf]

Additional file 1: List of primers used for qRT-PCR

PRIMER	SEQUENCE
RHOA-F<br>RHOA-R	5´-TGAAAACTATGTGGCGGATATCG-3'<br>5´-TCTGCTTCTTCAGGTTTAACCGG-3'
ITGB4-F<br>ITGB4-R	5´-GCGACTACACTATTGGATTTGGC-3'<br>5´-TGTCAGGCTGATGACGTTCTTG-3'
ITGA2-F<br>ITGA2-R	5´-TGCTGGTTGAAAGACGTTCACATG-3'<br>5´-TATAACTCCTGTTGGTACTTCGGC-3'
FAT2-F<br>FAT2-R	5´-CATGAATTCAAGCTGGATCCTC-3'<br>5´-CTCGGTCTAGGGCAGTGAGT-3'
PAK6-F<br>PAK6-R	5´-TGAGGAGCAGATTGCCACTGTG-3'<br>5´-CTGAGCACAGAATCCGAAGTCC-3'
DSC3-F<br>DSC3-R	5'-CACTTACTCGGAGTGGCACAGT-3'<br>5'-AGCTGGAGATCCTCTTCCCTCA-3'
MTSS1-F<br>MTSS1-R	5'-TCAAGAACAGATGGAAGAATGG-3'<br>5'-TGCGGTAGCGGTAATGTG-3'
CLDN1-F<br>CLDN1-R	5´-AATTCTATGACCCTATGACCC-3'<br>5´-GACAGGAACAGCAAAGTAGG-3'
KLK5-F<br>KLK5-R	5′-GCAGGTAGAGACTCCTGCCA-3'<br>5′-CACAAGGGTAATCTCCCCAG-3'
CLDN10-F<br>CLDN10-R	5´-TTGATCCTCTCTTTGTTGAGCA-3'<br>5´-AAGCAAAATATGACACCACCAA-3'
SVIL-F<br>SVIL-R	5´-TTCACTAAGAGCGGCAGAG-3'<br>5´-TTCTCCTGTGGCTGTTCC-3'
LAMA4-F<br>LAMA4-R	5´-GAGGAGGCAGATGAGGCTTAC-3'<br>5´-ACGACAGGAAACAGAGTGCGG-3'
CEACAM6-F<br>CEACAM6-R	5´-GAAATACAGAACCCAGCGAGTGC-3'<br>5´-CAGTGATGTTGGGGATAAAGAGC-3'
PAK1-F<br>PAK1-R	5´-GCTGTTCTGGATGTGTTGGA-3'<br>5´-TTCTGAAACTGGTGGCACTG-3'
VAV1-F<br>VAV1-R	5'-TGCTTCAAGTCTCTGGACACCAC-3'<br>5'-TCTCGGGCGCAGAAGTCATA-3'
PXN-F<br>PXN-R	5´-AACAAGCAGAAGTCAGCAGAGCC-3'<br>5´-CTAGCTTGTTCAGGTCGGAC-3'
RAB7B-F<br>RAB7B-R	5´-GGCCAGCATCCTCTCCAAGATTATC-3'<br>5´-GATGCAGCCATCGGAGCCCTTGT-3'
TNS3-F<br>TNS3-R	5´-GTTGAAAGGGTGCTCGAATGA-3'<br>5´-GAACTTTCTGCTATTTCCTCCAATG-3'
TNS4-F<br>TNS4-R	5´-CCCACCATGAAGTTCGTGATG-3'<br>5´-CGGTATGAAGAGCTGTCCCTTATG-3'
OLFM4-F	5´-AGCTCTTTCCCAGGTGTTGA-3'

OLFM4-R	5´-AAGCGTTCCACTCTGTCCAC-3'
LPXN-F<br>LPXN-R	5´-ACGCTCCACCCTTCAGGACA-3'<br>5´-GACATTGAGCTCCTGGATATTGG-3'
MUC1-F<br>MUC1-R	5´-CCTTTCTTCCTGCTGCTG-3'<br>5´-TGGGACTGAACTTCTCTG-3'
ΔNp63-F<br>ΔNp63-R	5’AGCCAGAAGAAAGGACAGCA’3<br>5’TCACTAAATTGAGTCTGGGCAT’3
TAp63-F<br>TAp63-R	5’GTCCCAGAGCACACAGACAA‘3<br>5’TGCGGATACAGTCCATGCTA‘3
