# Supplementary figures and images for: ΔNp63α expression induces loss of cell adhesion in triple-negative breast cancer cells
Source: BMC Cancer. 2016 Oct 10;16:782. doi: 10.1186/s12885-016-2808-x (PMC5057421; doi:10.1186/s12885-016-2808-x)

Additional file 2: Expression of *TP63* in breast cancer cell lines

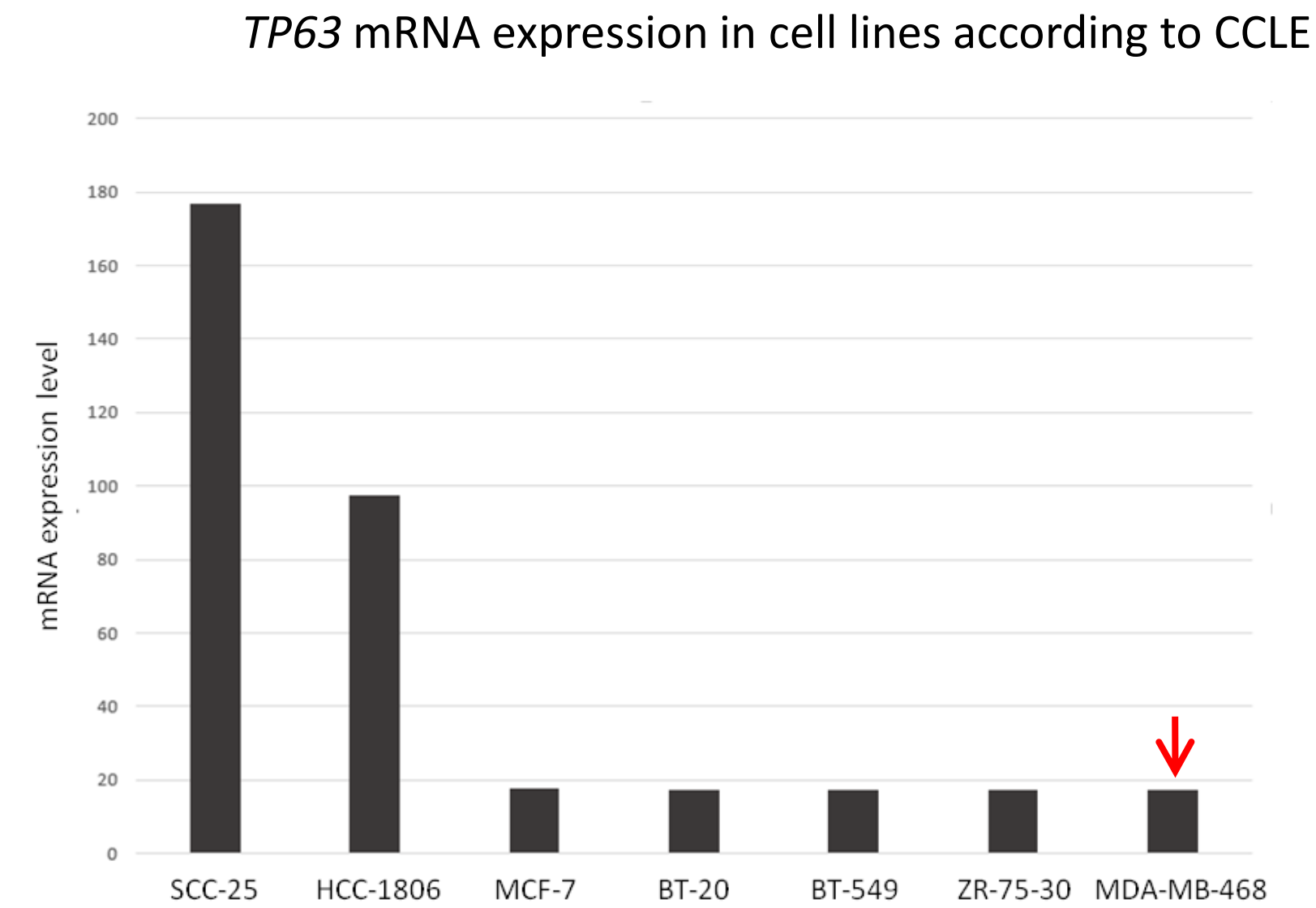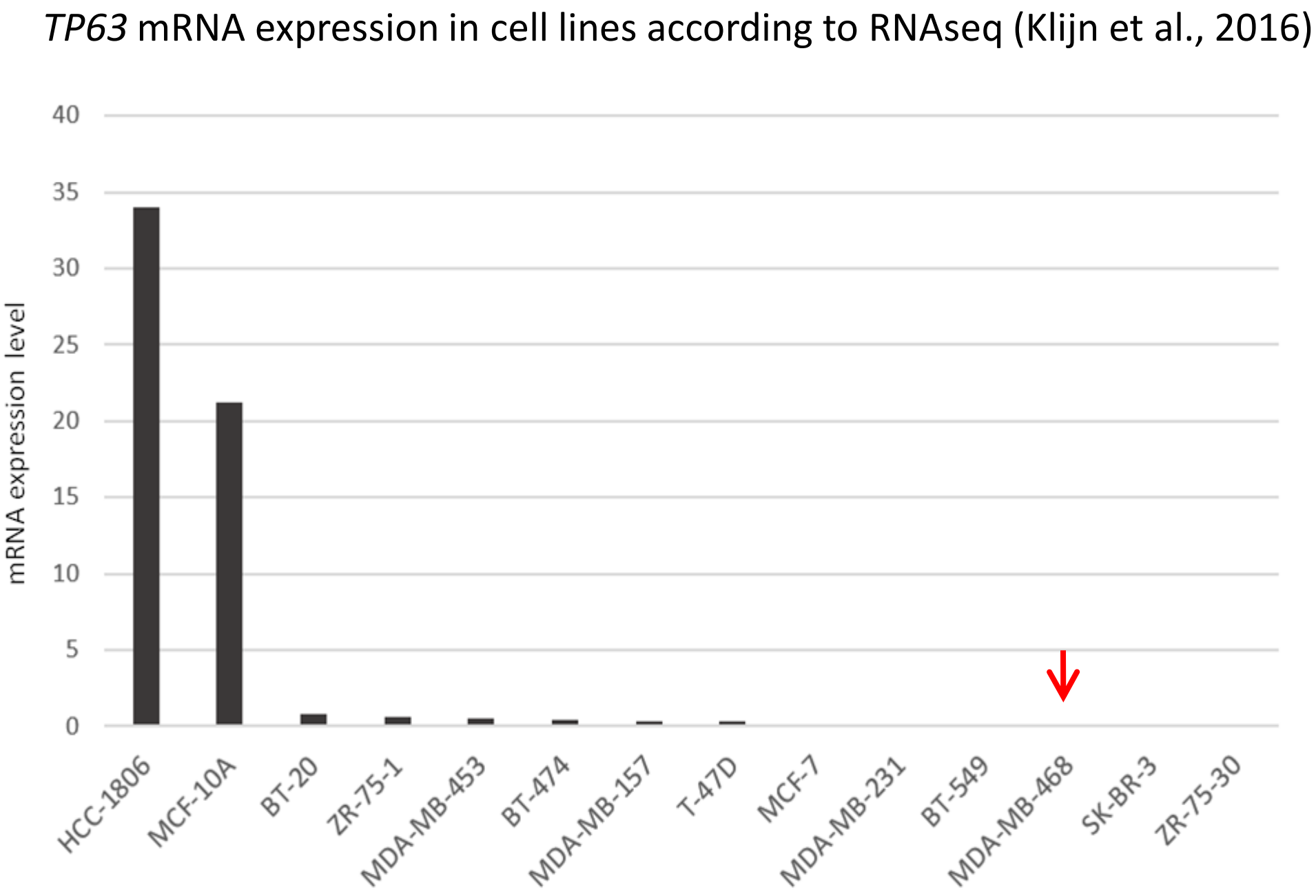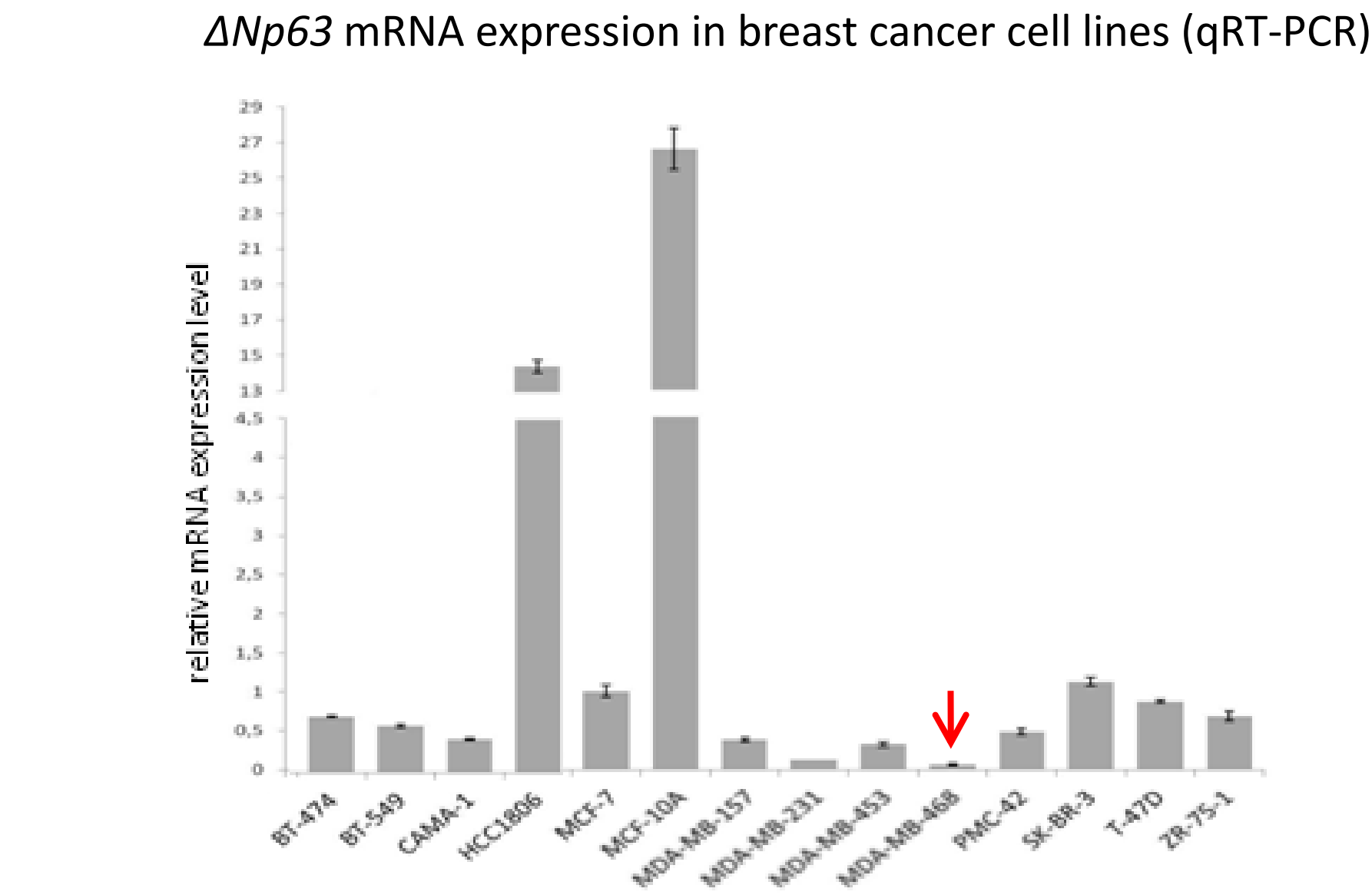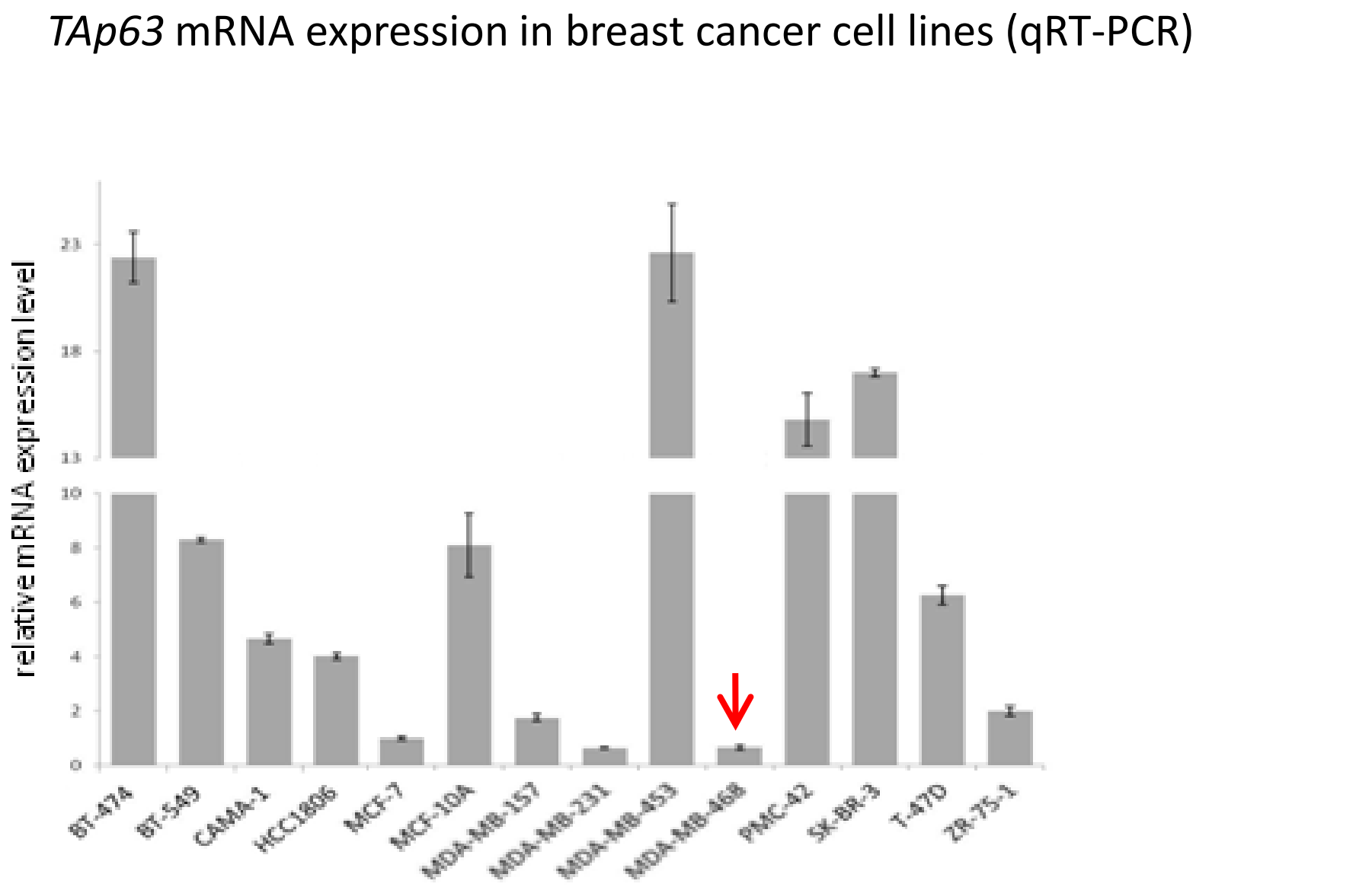

Supplement: Additional file 2: — Expression of TP63 in breast cancer cell lines. (PDF 407 kb) [file 12885_2016_2808_MOESM2_ESM.pdf]

**Additional file 4:** Measurement of anoikis resistance of MDA-MB-468- $\Delta$ Np63 $\alpha$  cells

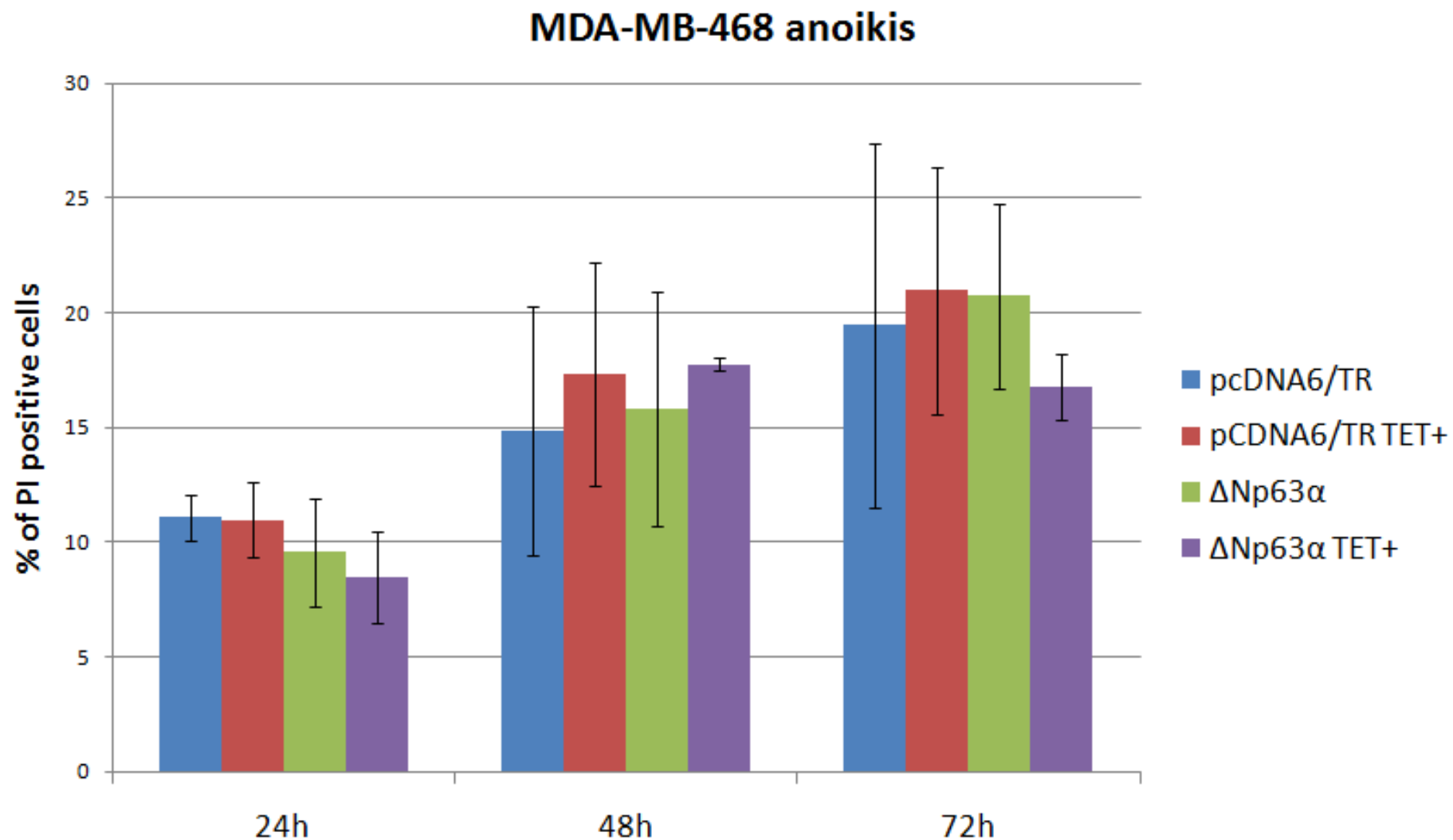

Supplement: Additional file 4: — Measurement of anoikis resistance of MDA-MB-468-∆Np63α cells. (PDF 249 kb) [file 12885_2016_2808_MOESM4_ESM.pdf]
