# Supplementary material for: ΔNp63α expression induces loss of cell adhesion in triple-negative breast cancer cells
Source: BMC Cancer. 2016 Oct 10;16:782. doi: 10.1186/s12885-016-2808-x (PMC5057421; doi:10.1186/s12885-016-2808-x)
Supplement: Additional file 3: — List of genes whose expression was changed 24 h after induction of ∆Np63α or TAp63α expression in MDA-MB-468 cells. (PDF 503 kb) [file 12885_2016_2808_MOESM3_ESM.pdf]

Additional file 3: List of genes whose expression was changed 24 h after induction of ΔNp63α or TAp63α expression in MDA-MB-468 cells.

Genes differentially expressed 24 h after induction of ΔNp63α expression in MDA-MB-468-ΔNp63α cells ($p < 0.05$)			
**PROBE_ID**	**GENE SYMBOL**	**adj-p-values,**	**fold change**
ILMN_3224962	TP63	7.2E-07	13.5
ILMN_1771688	RAB7B	2.0E-05	7.5
ILMN_1725852	S100A2	4.6E-07	4.8
ILMN_1723123	FGFR3	1.1E-05	4.5
ILMN_1690454	C3orf54	9.9E-05	4.2
ILMN_1752755	VWF	1.8E-04	3.8
ILMN_1696585	FERMT1	2.2E-04	3.7
ILMN_2094266	HES2	8.6E-05	3.7
ILMN_2133205	GPX2	7.1E-05	3.6
ILMN_2073184	S1PR5	1.5E-03	3.6
ILMN_1656422	GPR87	1.1E-05	3.1
ILMN_1666076	ARHGEF4	7.7E-05	3.0
ILMN_1758613	RAPGEFL1	7.7E-05	3.0
ILMN_1741755	TRIM29	1.5E-04	3.0
ILMN_1758888	PADI3	2.1E-04	2.9
ILMN_2134974	RAB38	1.8E-04	2.9
ILMN_2132599	ANKRD22	2.8E-05	2.9
ILMN_2128931	FAT2	1.1E-05	2.8
ILMN_2334210	ITGB4	1.8E-04	2.7
ILMN_1735365	GJB5	3.1E-04	2.7
ILMN_2228162	KRT16	5.4E-03	2.6
ILMN_3308295	MIR205	1.3E-02	2.6
ILMN_2219002	KRT6A	1.1E-02	2.5
ILMN_1666845	KRT17	1.1E-05	2.5
ILMN_1708223	PAK6	9.8E-04	2.4
ILMN_1764571	ARHGAP23	7.8E-05	2.3
ILMN_1777998	ARHGAP25	8.6E-03	2.3
ILMN_2072357	IRF6	4.2E-04	2.3
ILMN_1738558	RGS20	6.7E-04	2.3
ILMN_1738742	PLAT	2.6E-03	2.2
ILMN_1784602	CDKN1A	6.5E-03	2.2
ILMN_2339835	PTGS1	3.6E-03	2.2
ILMN_1765363	DSC3	9.6E-04	2.2
ILMN_2148469	RASL11B	9.7E-03	2.2
ILMN_1693218	ART3	7.3E-04	2.2
ILMN_1777170	LOC440585	9.7E-03	2.1
ILMN_1782863	FAM83B	5.3E-03	2.1
ILMN_1665792	ITGA2	8.6E-05	2.1
ILMN_1799098	LOC652846	1.1E-03	2.1

ILMN_2073289	MTSS1	1.1E-02	2.1
ILMN_1791545	KRT23	1.8E-02	2.1
ILMN_2186983	ANXA8L2	1.9E-04	2.1
ILMN_1653934	LOC650517	4.5E-04	2.1
ILMN_2095610	ANXA8	8.8E-03	2.1
ILMN_1700583	ZNF750	6.7E-03	2.1
ILMN_1665865	IGFBP4	2.9E-03	2.1
ILMN_1652185	IL4R	9.2E-04	2.1
ILMN_2134056	SOX7	9.3E-05	2.1
ILMN_1770612	KRT15	6.4E-04	2.0
ILMN_2221046	GM2A	3.9E-02	2.0
ILMN_1654217	MPP2	7.0E-03	2.0
ILMN_1693090	CROT	4.9E-03	2.0
ILMN_1739605	LYPD3	2.9E-03	2.0
ILMN_1794501	HAS3	1.0E-02	2.0
ILMN_1720158	ETS2	1.9E-03	2.0
ILMN_1808707	FSCN1	1.3E-03	1.9
ILMN_1724686	CLDN1	1.2E-04	1.9
ILMN_1723418	CEL	7.0E-03	1.9
ILMN_1661628	LOC653110	1.1E-03	1.9
ILMN_1806607	SFN	2.2E-04	1.9
ILMN_1715647	VANGL2	2.7E-03	1.9
ILMN_3195198	KRT17P3	2.9E-04	1.9
ILMN_2189027	LIPG	4.5E-02	1.9
ILMN_1684211	SEC14L2	4.7E-03	1.9
ILMN_2095759	OGFRL1	2.0E-03	1.9
ILMN_1792885	CTSC	6.4E-03	1.9
ILMN_1659766	BAG3	1.8E-03	1.9
ILMN_1718731	KLK5	2.9E-02	1.9
ILMN_2334531	GJB3	4.9E-03	1.8
ILMN_1661137	SH3RF2	7.0E-03	1.8
ILMN_1656938	LOC731486	3.1E-03	1.8
ILMN_1669888	CSTA	1.8E-03	1.8
ILMN_1659106	PHLDA3	5.3E-03	1.8
ILMN_1752932	MPZL2	1.9E-03	1.8
ILMN_1801632	KRT5	5.7E-03	1.8
ILMN_1719883	CYP4F11	5.1E-03	1.8
ILMN_1695959	C21orf63	2.9E-03	1.8
ILMN_1671123	LOC647543	1.6E-03	1.7
ILMN_1684653	GPR115	2.8E-02	1.7
ILMN_1703435	ZNF658	2.5E-02	1.7
ILMN_1729161	NOTCH1	1.4E-03	1.7
ILMN_1780132	PELI2	1.3E-02	1.7
ILMN_1761820	EDARADD	1.9E-02	1.7
ILMN_1676413	VSNL1	5.4E-03	1.7
ILMN_2104106	XPR1	9.2E-04	1.7

ILMN_2098418	LOC652968	3.6E-03	1.7
ILMN_1708041	PLEKHF1	7.2E-03	1.7
ILMN_1741544	KRT38	4.9E-03	1.7
ILMN_1737988	PRNP	1.6E-03	1.7
ILMN_1670325	SLC47A1	4.2E-03	1.7
ILMN_1803236	CLCA2	8.3E-03	1.6
ILMN_2124471	SLC36A1	1.0E-02	1.6
ILMN_1733045	RAB36	4.1E-04	1.6
ILMN_1751034	ITPRIPL2	2.6E-02	1.6
ILMN_1794825	ALDH3A2	4.5E-04	1.6
ILMN_1815130	MICALL1	4.3E-04	1.6
ILMN_1803256	STOX2	5.0E-02	1.6
ILMN_1778144	FLJ20489	2.0E-02	1.6
ILMN_1769849	HORMAD1	4.8E-02	1.6
ILMN_1654735	SLCO3A1	2.7E-03	1.6
ILMN_1699809	CAPNS2	8.5E-03	1.6
ILMN_2088124	TMEM154	5.6E-03	1.6
ILMN_1699489	TUBB6	6.2E-04	1.6
ILMN_1743103	SH3PXD2A	2.0E-02	1.6
ILMN_3242004	ANXA8L1	2.5E-03	1.6
ILMN_2332250	ACOT7	4.9E-03	1.6
ILMN_1702787	SEMA4A	2.7E-02	1.5
ILMN_1686679	ZNF462	3.8E-03	1.5
ILMN_2382290	KREMEN2	2.4E-02	1.5
ILMN_1758146	SIRPA	3.2E-03	1.5
ILMN_1723412	ASCL2	3.4E-02	1.5
ILMN_3243924	P2RX6	3.0E-03	1.5
ILMN_2052208	GADD45A	3.4E-02	1.5
ILMN_1748881	MRAS	4.5E-02	1.5
ILMN_2219246	LOC162073	4.8E-02	1.5
ILMN_1664861	ID1	4.5E-03	1.5
ILMN_3235221	LOC644936	5.7E-03	1.5
ILMN_1698732	PALLD	4.8E-02	1.5
ILMN_1752451	CTSH	1.8E-02	1.5
ILMN_1737208	NTF5	8.3E-03	1.5
ILMN_2329679	TPST2	7.1E-03	1.5
ILMN_1803647	FAM162A	4.8E-02	1.5
ILMN_1786326	KCTD15	2.3E-02	1.5
ILMN_2138589	MERTK	7.0E-03	1.5
ILMN_1675612	BLCAP	1.9E-02	1.5
ILMN_1766054	ABCA1	1.0E-02	1.5
ILMN_1769546	RIN2	5.7E-03	1.5
ILMN_1676665	CUEDC1	5.9E-03	1.5
ILMN_1721818	CLDN10	1.2E-02	1.5
ILMN_1689585	C20orf194	1.9E-02	1.5
ILMN_1765607	SLC6A2	4.9E-02	1.5

ILMN_1664267	WBSCR28	1.9E-02	1.5
ILMN_1671404	SVIL	1.0E-03	1.5
ILMN_1744381	SERPINE1	2.9E-02	1.4
ILMN_3298400	LOC731954	2.1E-02	1.4
ILMN_2396546	IGSF3	7.6E-04	1.4
ILMN_2063114	TAF1D	3.8E-02	1.4
ILMN_1756784	FREQ	1.2E-02	1.4
ILMN_1673769	KCNG1	1.4E-02	1.4
ILMN_2153485	NMNAT3	1.2E-02	1.4
ILMN_1761463	EFHD2	2.3E-03	1.4
ILMN_1664698	UNC119	2.0E-02	1.4
ILMN_3247723	NOP16	9.5E-03	1.4
ILMN_2096719	GRK5	1.3E-02	1.4
ILMN_1788942	GGT6	1.8E-02	1.4
ILMN_1712046	CPXM1	2.9E-02	1.4
ILMN_1809894	TMEM117	2.7E-02	1.4
ILMN_1793859	ALDH2	4.8E-02	1.4
ILMN_3227263	SLC22A23	9.2E-03	1.4
ILMN_1785404	FGFBP1	9.2E-03	1.4
ILMN_1804539	GPR89B	1.7E-02	1.4
ILMN_1651819	GALNT11	6.6E-03	1.4
ILMN_2048636	ME2	1.4E-02	1.4
ILMN_1669557	AIM1L	4.7E-02	1.4
ILMN_2181241	LOC649946	4.8E-02	1.4
ILMN_1721559	FABP6	2.1E-02	1.4
ILMN_1667239	INPP1	4.5E-02	1.4
ILMN_2224657	KLRG2	1.5E-02	1.4
ILMN_1795865	FGFRL1	2.4E-02	1.4
ILMN_1809099	IL33	2.7E-02	1.3
ILMN_1716370	TNS4	3.5E-02	1.3
ILMN_1660976	LOC653204	3.1E-02	1.3
ILMN_1772123	ACACA	9.9E-03	1.3
ILMN_1705116	C6orf85	2.2E-02	1.3
ILMN_1654385	ASB13	1.9E-02	1.3
ILMN_1783304	ATP1B3	3.2E-02	1.3
ILMN_3199755	LOC646821	2.9E-02	1.3
ILMN_1792660	CAMSAP1L1	1.2E-02	1.3
ILMN_2311537	HMGA1	2.6E-03	1.3
ILMN_1687275	JUB	4.1E-03	1.3
ILMN_2357781	ZNF436	7.7E-03	1.3
ILMN_2147517	CD58	3.6E-02	1.3
ILMN_1659027	SLC2A1	5.4E-03	1.3
ILMN_1729749	HERC5	2.5E-02	1.3
ILMN_2362902	RASSF5	9.9E-03	1.3
ILMN_2361400	ABCA12	2.1E-02	1.3
ILMN_1710209	MFSD6	4.8E-02	1.3

ILMN_1745954	CORO1C	2.0E-02	1.3
ILMN_2359211	AP2A1	3.1E-02	1.3
ILMN_1764090	AK3L1	2.9E-02	1.3
ILMN_1690826	TNKS1BP1	1.8E-02	1.3
ILMN_3287266	LOC100133328	3.5E-02	1.3
ILMN_1696316	CPT1A	2.7E-02	1.3
ILMN_3236765	UPLP	4.8E-02	1.3
ILMN_1777499	LOC731007	2.1E-02	1.3
ILMN_1792182	RDH12	1.5E-02	1.3
ILMN_1775677	TYSND1	1.3E-02	1.3
ILMN_1739001	TACSTD2	1.2E-02	1.3
ILMN_2353202	PTK7	2.4E-02	1.3
ILMN_1740170	CHCHD10	3.2E-02	1.3
ILMN_1780298	FAM86A	4.4E-02	1.3
ILMN_1688295	ZNF219	3.1E-02	1.3
ILMN_1714667	C20orf107	1.1E-02	1.3
ILMN_2184640	NOLC1	4.5E-02	1.2
ILMN_2142353	GRTP1	3.1E-02	1.2
ILMN_2386008	MPZL1	3.5E-02	1.2
ILMN_2184184	ANXA1	4.9E-02	1.2
ILMN_1666306	SRRD	4.6E-02	1.2
ILMN_1800465	LOC654042	4.1E-02	0.8
ILMN_1735093	TIMELESS	2.6E-02	0.8
ILMN_1667101	PRM3	2.6E-02	0.8
ILMN_1703886	SLC16A2	2.9E-02	0.8
ILMN_3176989	HAUS8	4.9E-02	0.8
ILMN_2399769	GPR177	4.8E-02	0.8
ILMN_1752728	FUCA1	2.0E-02	0.8
ILMN_3288755	LOC646808	4.6E-02	0.8
ILMN_1734190	TCEAL3	5.4E-03	0.8
ILMN_1672148	AKR1B10	2.0E-02	0.8
ILMN_1784110	PCTK3	4.7E-02	0.8
ILMN_2374778	DUT	9.2E-03	0.8
ILMN_1682336	MASTL	2.9E-02	0.8
ILMN_2230892	IL10RB	1.8E-02	0.8
ILMN_1700633	ABHD4	1.2E-02	0.8
ILMN_3248091	C6orf223	4.6E-02	0.8
ILMN_1751097	CREB3L2	1.5E-02	0.8
ILMN_1668453	TRIM36	2.7E-02	0.8
ILMN_1763091	C14orf43	1.2E-02	0.8
ILMN_1694780	GCHFR	3.2E-02	0.8
ILMN_1666924	PINK1	9.1E-03	0.8
ILMN_1662852	IQCK	2.6E-02	0.8
ILMN_2340347	PC	3.8E-02	0.8
ILMN_1779855	HSD17B6	4.8E-02	0.8
ILMN_1666109	MB	4.7E-02	0.8

ILMN_1654331	HOXB4	3.8E-02	0.8
ILMN_1774110	CHN2	4.9E-02	0.8
ILMN_1715789	DOCK1	3.0E-02	0.8
ILMN_1684336	VEPH1	3.5E-02	0.8
ILMN_1758392	ANKS1B	3.3E-02	0.8
ILMN_1781691	TRAK2	2.9E-02	0.8
ILMN_2139761	LIMCH1	1.4E-02	0.8
ILMN_1779015	ZNF467	3.8E-02	0.8
ILMN_1680037	FAM65A	4.8E-02	0.8
ILMN_2057573	FAM62B	3.2E-02	0.8
ILMN_2364110	GBA	2.6E-02	0.8
ILMN_1677292	C5orf30	3.6E-02	0.8
ILMN_1755111	C6orf199	4.4E-02	0.8
ILMN_1775268	HECW2	1.8E-02	0.8
ILMN_2052373	RAB17	2.3E-02	0.8
ILMN_1678454	CASP4	4.6E-02	0.8
ILMN_1746359	RERG	1.3E-02	0.8
ILMN_1811006	E2F8	3.3E-02	0.8
ILMN_2338323	CDC25B	3.1E-02	0.8
ILMN_1663092	CITED2	3.0E-02	0.8
ILMN_1653711	FZD2	4.9E-02	0.7
ILMN_1794863	CAMK2N1	3.5E-02	0.7
ILMN_1742789	LPXN	2.9E-02	0.7
ILMN_2060115	SORL1	9.3E-03	0.7
ILMN_1670028	LPIN2	3.1E-02	0.7
ILMN_1688811	SPRY4	4.9E-02	0.7
ILMN_1690703	C21orf34	4.8E-02	0.7
ILMN_1685580	CBLB	4.9E-02	0.7
ILMN_1658709	LAMB1	4.4E-02	0.7
ILMN_1752927	KIAA1600	1.9E-02	0.7
ILMN_3231638	FAM160B1	7.5E-03	0.7
ILMN_1725726	DHRS2	3.5E-02	0.7
ILMN_1795247	ARID2	5.3E-03	0.7
ILMN_1704286	FXYD5	7.7E-03	0.7
ILMN_1763322	CCR3	4.4E-02	0.7
ILMN_1677814	ABCC3	1.0E-02	0.7
ILMN_1809077	RFPL4B	2.2E-02	0.7
ILMN_1769925	C2CD4A	4.9E-02	0.7
ILMN_1751234	C1GALT1C1	1.0E-02	0.7
ILMN_1736757	GNPTAB	4.1E-02	0.7
ILMN_1795704	KIAA0232	1.3E-02	0.7
ILMN_1665964	GAB2	5.0E-02	0.7
ILMN_1753010	PET112L	1.9E-03	0.7
ILMN_2178226	KRT86	1.2E-02	0.7
ILMN_1720996	SLC12A2	4.3E-02	0.7
ILMN_1752510	FAM13A	3.8E-02	0.7

ILMN_1723522	APOLD1	4.7E-02	0.7
ILMN_1673409	MGC16121	1.0E-02	0.7
ILMN_1651496	HIST1H2BD	5.6E-03	0.7
ILMN_1716265	PGM2L1	1.9E-02	0.7
ILMN_1800091	RARRES1	1.1E-02	0.7
ILMN_1791123	TMPRSS2	4.8E-02	0.7
ILMN_1733937	MMD	1.2E-02	0.7
ILMN_1746952	LOC153328	4.7E-02	0.7
ILMN_1723962	LXN	2.7E-02	0.7
ILMN_1708375	IRF1	2.3E-02	0.7
ILMN_1777644	PIB5PA	6.4E-04	0.7
ILMN_1779448	EFHD1	3.4E-03	0.7
ILMN_1737514	KYNU	9.2E-03	0.7
ILMN_1687538	ETS1	8.0E-03	0.7
ILMN_1776936	ANKRD38	1.2E-02	0.7
ILMN_1690695	PEX11A	4.5E-02	0.7
ILMN_1678692	MPRIP	4.9E-03	0.7
ILMN_1698725	FRMD3	2.9E-02	0.7
ILMN_1709882	ICK	1.8E-02	0.7
ILMN_1671971	LOC644743	2.9E-03	0.7
ILMN_1753135	ALK	3.5E-02	0.7
ILMN_2371911	MUC1	2.1E-02	0.7
ILMN_1705080	SLURP1	1.8E-02	0.7
ILMN_1803423	ARHGEF6	9.1E-03	0.7
ILMN_1695290	FERMT2	1.1E-02	0.7
ILMN_1709683	RASSF2	1.7E-02	0.7
ILMN_1665331	AMT	1.2E-02	0.7
ILMN_2353633	EMR2	4.3E-02	0.7
ILMN_1705984	HNMT	6.9E-03	0.7
ILMN_1779252	TRIM22	4.8E-02	0.7
ILMN_1742332	KCTD12	2.5E-02	0.7
ILMN_2301722	PDE8B	7.0E-03	0.7
ILMN_2068104	TFPI2	2.5E-02	0.7
ILMN_1753584	KRT8	8.7E-03	0.7
ILMN_2257607	STARD13	1.2E-02	0.7
ILMN_1774901	GDPD3	2.9E-02	0.7
ILMN_1758272	MYPN	3.3E-03	0.7
ILMN_1724598	RABL2A	2.1E-02	0.7
ILMN_1712431	FAM113B	7.1E-03	0.7
ILMN_1659836	RAET1G	4.8E-02	0.7
ILMN_1691156	MT1A	2.7E-02	0.7
ILMN_1700042	TLN2	9.3E-03	0.7
ILMN_1687867	LOC647954	2.6E-02	0.7
ILMN_1698038	FAM188B	5.6E-03	0.7
ILMN_1768004	PDCD4	3.9E-02	0.7
ILMN_1694458	FLJ41200	3.1E-02	0.7

ILMN_1759550	LOC644086	2.2E-02	0.7
ILMN_2113490	NTN4	2.1E-02	0.7
ILMN_1815610	SYT12	3.1E-03	0.7
ILMN_1711904	MXD3	1.3E-02	0.7
ILMN_1681737	TMSB15A	4.5E-02	0.7
ILMN_1736911	TMOD1	3.6E-03	0.7
ILMN_1660552	BMP5	1.1E-02	0.7
ILMN_1796094	CD36	6.1E-03	0.7
ILMN_2228463	DDC	1.6E-03	0.7
ILMN_2159290	LOC441376	2.7E-02	0.7
ILMN_1658639	SLC46A3	1.7E-04	0.7
ILMN_1655904	ROR1	5.3E-03	0.6
ILMN_1750373	KAL1	2.5E-02	0.6
ILMN_1811272	GPR81	9.8E-03	0.6
ILMN_1662619	TFPI	1.1E-03	0.6
ILMN_1659856	FAM167B	3.6E-02	0.6
ILMN_1752478	DHRS3	6.5E-03	0.6
ILMN_1810420	DYSF	2.4E-02	0.6
ILMN_1688580	CAMP	8.8E-03	0.6
ILMN_1810191	PLA2G4C	2.6E-03	0.6
ILMN_1684497	C10orf33	2.6E-02	0.6
ILMN_1802654	GLT8D2	3.6E-02	0.6
ILMN_1744118	ASTN2	2.3E-02	0.6
ILMN_1801216	S100P	5.3E-03	0.6
ILMN_2190541	XAGE2B	7.9E-03	0.6
ILMN_1724181	IL15	7.0E-03	0.6
ILMN_1774604	PNKD	4.4E-02	0.6
ILMN_1747227	ADORA1	9.7E-03	0.6
ILMN_1746801	CGN	2.9E-03	0.6
ILMN_1734542	OVGP1	2.1E-02	0.6
ILMN_1703374	NAV1	5.3E-03	0.6
ILMN_1753507	FRMPD3	2.6E-02	0.6
ILMN_1781966	OSBP2	1.2E-04	0.6
ILMN_1730117	TMC5	1.9E-03	0.6
ILMN_1686664	MT2A	8.5E-03	0.6
ILMN_1693192	PI3	1.2E-02	0.6
ILMN_1785071	SEPP1	1.8E-03	0.6
ILMN_1755354	YBX2	1.1E-03	0.6
ILMN_1736184	GSTM3	4.9E-03	0.6
ILMN_1786041	ASB9	1.4E-02	0.6
ILMN_1775285	ATP13A5	6.9E-03	0.6
ILMN_1684377	LOC649030	4.5E-03	0.6
ILMN_1723443	LRP2	3.7E-03	0.6
ILMN_1658071	ATP1B1	1.9E-02	0.6
ILMN_1682181	PHKG1	1.1E-02	0.6
ILMN_1735816	CYP4A11	1.2E-02	0.6

ILMN_1777853	MBOAT2	1.8E-02	0.6
ILMN_1748751	NLF2	1.1E-02	0.6
ILMN_1678170	MME	4.8E-02	0.6
ILMN_1774077	GBP2	2.3E-03	0.6
ILMN_2054121	C6orf126	2.9E-02	0.6
ILMN_1674817	C1orf115	1.5E-04	0.6
ILMN_2071809	MGP	3.6E-03	0.6
ILMN_1712305	CYBRD1	3.2E-02	0.6
ILMN_1778956	STS	2.0E-02	0.6
ILMN_1680652	SELENBP1	1.1E-03	0.6
ILMN_1695590	ADRB2	2.0E-04	0.6
ILMN_1718852	PLCL1	1.0E-02	0.6
ILMN_2371280	CSF3R	4.8E-02	0.6
ILMN_1669338	CYP4X1	5.1E-03	0.6
ILMN_1699574	NRP1	2.0E-03	0.6
ILMN_1723139	GPD2	2.1E-02	0.6
ILMN_1691410	BAMBI	5.7E-03	0.6
ILMN_1746676	CLDN8	5.6E-03	0.6
ILMN_1766499	HSPA2	1.5E-03	0.6
ILMN_1771385	GBP4	2.8E-02	0.6
ILMN_1709067	SAMD11	5.1E-03	0.6
ILMN_1761903	KCNS1	1.3E-03	0.6
ILMN_2376403	TSC22D3	2.9E-03	0.5
ILMN_1697491	FLJ14213	3.5E-02	0.5
ILMN_1664176	FBLN5	2.9E-02	0.5
ILMN_1741768	TMPRSS3	2.7E-05	0.5
ILMN_1685608	NPTX2	2.9E-03	0.5
ILMN_2136446	CTNNAL1	4.2E-04	0.5
ILMN_1784447	PLCE1	3.4E-02	0.5
ILMN_1807652	STRA6	1.8E-03	0.5
ILMN_1723333	SCGB2A2	5.9E-03	0.5
ILMN_1729417	GNE	1.3E-02	0.5
ILMN_2380237	C1QTNF1	4.9E-03	0.5
ILMN_1697220	NT5E	3.4E-02	0.5
ILMN_2410612	DMBT1	1.2E-02	0.5
ILMN_1803528	LRRC31	2.1E-03	0.5
ILMN_1749792	SORBS1	3.1E-02	0.5
ILMN_1712522	CEACAM6	1.8E-03	0.5
ILMN_2343097	NCALD	1.6E-03	0.5
ILMN_2394287	ATP6V0A4	2.1E-04	0.5
ILMN_1714335	RDH10	5.7E-03	0.5
ILMN_1695354	BMF	1.6E-03	0.5
ILMN_3290136	LOC643733	1.4E-02	0.5
ILMN_2116299	GRRP1	2.2E-03	0.5
ILMN_1675808	TCHHL1	9.2E-04	0.5
ILMN_1714536	SCGB1D2	1.1E-03	0.5

ILMN_1740900	BMP4	2.3E-02	0.5
ILMN_1669376	DRAM1	1.2E-04	0.5
ILMN_1812073	ATP6V1B1	4.0E-04	0.5
ILMN_1678690	UPB1	7.1E-04	0.5
ILMN_2340259	PDE4B	8.3E-04	0.4
ILMN_2088437	CX3CR1	7.2E-03	0.4
ILMN_3194638	FAM176A	5.9E-04	0.4
ILMN_1795442	LAMA4	7.7E-05	0.4
ILMN_1702829	CYP4Z2P	1.8E-04	0.4
ILMN_1702973	TMEM166	2.8E-04	0.4
ILMN_2116877	OLFM4	2.8E-03	0.4
ILMN_1757099	C10orf81	6.9E-03	0.4
ILMN_1732398	SCGB2A1	1.2E-04	0.4
ILMN_1662795	CA2	3.2E-03	0.4
ILMN_1728550	CYP4Z1	4.3E-04	0.4
ILMN_1716859	TDO2	2.0E-04	0.4
ILMN_1704554	LOC648470	9.5E-05	0.4
ILMN_1737650	DIO2	1.1E-05	0.3
ILMN_1667893	TNS3	1.1E-05	0.3
ILMN_2161820	GLYATL2	6.1E-05	0.3

Genes differentially expressed 24 h after induction of TAp63α expression in MDA-MB-468-TAp63α cells ($p < 0.05$)			
**PROBE_ID**	**GENE SYMBOL**	**adj-p-values**	**fold change**
ILMN_3224962	TP63	5.3E-06	10.2
ILMN_2132599	ANKRD22	4.2E-02	1.9
